# 3D-printed cell-free PCL–MECM scaffold with biomimetic micro-structure and micro-environment to enhance in situ meniscus regeneration

**DOI:** 10.1016/j.bioactmat.2021.02.019

**Published:** 2021-03-27

**Authors:** Weimin Guo, Mingxue Chen, Zhenyong Wang, Yue Tian, Jinxuan Zheng, Shuang Gao, Yangyang Li, Yufeng Zheng, Xu Li, Jingxiang Huang, Wei Niu, Shuangpeng Jiang, Chunxiang Hao, Zhiguo Yuan, Yu Zhang, Mingjie Wang, Zehao Wang, Jiang Peng, Aiyuan Wang, Yu Wang, Xiang Sui, Wenjing Xu, Libo Hao, Xifu Zheng, Shuyun Liu, Quanyi Guo

**Affiliations:** a Department of Orthopaedic Surgery, First Affiliated Hospital, Sun Yat-sen University, No.58 Zhongshan Second Road, Yuexiu District, Guangzhou, Guangdong, 510080, People's Republic of China; b Institute of Orthopedics, The First Medical Center, Chinese PLA General Hospital; Beijing Key Lab of Regenerative Medicine in Orthopedics, Key Laboratory of Musculoskeletal Trauma & War Injuries PLA. No.28 Fuxing Road, Haidian District, Beijing 100853, People's Republic of China; c Department of Orthopedic Surgery, Beijing Jishuitan Hospital, Fourth Clinical College of Peking University, No. 31 Xinjiekou East Street, Xicheng District, Beijing, 100035, People's Republic of China; d Department of Orthodontics, Guanghua School of Stomatology, Guangdong Provincial Key Laboratory of Stomatology, Sun Yat-sen University, No.56 Linyuan Xi Road, Yuexiu District, Guangzhou, Guangdong 510055, People's Republic of China; e Center for Biomaterial and Tissue Engineering, Academy for Advanced Interdisciplinary Studies, Peking University, Beijing, 100871, People's Republic of China; f Department of Materials Science and Engineering, College of Engineering, Peking University, Beijing 100871*,* People's Republic of China; g Musculoskeletal Research Laboratory, Department of Orthopaedics and Traumatology, Innovative Orthopaedic Biomaterial and Drug Translational Research Laboratory, Li Ka Shing Institute of Health Sciences, The Chinese University of Hong Kong, Hong Kong, People's Republic of China; h Institute of Anesthesiology, The First Medical Center, Chinese PLA General Hospital, No. 28 Fuxing Road, Haidian District, Beijing 100853, People's Republic of China; i Department of Orthopedic Surgery, First Affiliated Hospital, Dalian Medical University, No. 222 Zhongshan Road, Xigang District, Dalian 116011, China

**Keywords:** 3D printing, Cell-free, Decellularization, Biomimetic scaffold, Meniscus regeneration

## Abstract

Despite intensive effort was made to regenerate injured meniscus by cell-free strategies through recruiting endogenous stem/progenitor cells, meniscus regeneration remains a great challenge in clinic. In this study, we found decellularized meniscal extracellular matrix (MECM) preserved native meniscal collagen and glycosaminoglycans which could be a good endogenous regeneration guider for stem cells. Moreover, MECM significantly promoted meniscal fibrochondrocytes viability and proliferation, increased the expression of type II collagen and proteoglycans in vitro. Meanwhile, we designed 3D-printed polycaprolactone (PCL) scaffolds which mimic the circumferential and radial collagen orientation in native meniscus. Taken these two advantages together, a micro-structure and micro-environment dually biomimetic cell-free scaffold was manipulated. This cell-free PCL-MECM scaffold displayed superior biocompatibility and yielded favorable biomechanical capacities closely to native meniscus. Strikingly, neo-menisci were regenerated within PCL-MECM scaffolds which were transplanted into knee joints underwent medial meniscectomy in rabbits and sheep models. Histological staining confirmed neo-menisci showed meniscus-like heterogeneous staining. Mankin scores showed PCL-MECM scaffold could protect articular cartilage well, and knee X-ray examination revealed same results. Knee magnetic resonance imaging (MRI) scanning also showed some neo-menisci in PCL-MECM scaffold group. In conclusion, PCL-MECM scaffold appears to optimize meniscus regeneration. This could represent a promising approach worthy of further investigation in preclinical applications.

## Highlights

• 3D-printed PCL scaffolds could mimic the circumferential and radial collagen orientation in native meniscus.
• PCL-MECM scaffold displayed superior biocompatibility and yielded favorable biomechanical capacities.
• PCL-MECM scaffold appears to optimize meniscus regeneration in both rabbit and sheep meniscus repairing model.
• PCL-MECM scaffold may represent a promising approach worthy of further investigation in preclinical applications.

## Graphical abstract

**Figure:**
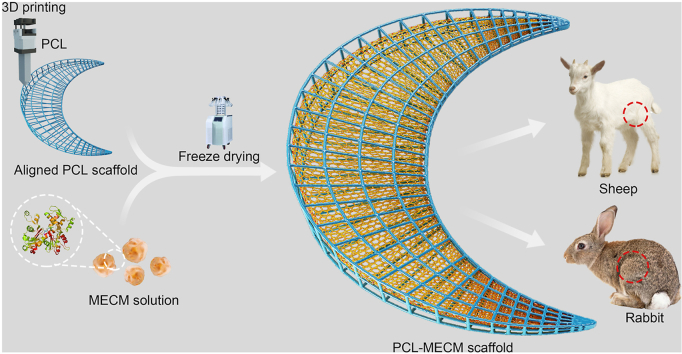

## Abbreviation

MECM: Meniscal extracellular matrix
PCL: Polycaprolactone
FDA: Food and Drug Administration
ECM: Extracellular matrix
PBS: Phosphate-buffered saline
rpm: Revolutions per minute
RT: Room temperature
sGAG: Sulfated glycosaminoglycans
CS: Chondroitin sulfate
TCP: The control group
AFM: Atomic force microscopy
SEM: Scanning electron microscopy
DMEM: Dulbecco's modified Eagle's medium
DAB: Diaminobenzidine tetrahydrochloride
LA: Large pore/aligned
LU: Large pore/unaligned
SA: Small pore/aligned
SU: Small pore/unaligned
EDAC: Carbodiimide hydrochloride
DMMB: 1,9-dimethylmethylene blue
WORMS: Whole-Organ Magnetic Resonance Imaging Scores
EDTA: Ethylene diamine tetraacetic acid
SD: Standard deviation
ANOVA: Analysis of variance
RMS: Root mean square
GFP: Green fluorescent protein
CMI®: Collagen Meniscus Implant
MRI: Magnetic resonance imaging
HE: Hexamethyldisilazane

## 1 Introduction

The menisci are a pair of wedge-shaped fibrocartilaginous tissues located in the knee joint that serve critical functions in load transmission, shock absorption, joint stabilization and lubrication, as well as joint health. Menisci are commonly injured by trauma or degenerative changes [1]. An injured meniscus is closely associated with an increased risk of knee osteoarthritis, followed by considerable pain and discomfort [2]. For the peripheral vertical meniscus tears, suture repair is the primarily therapeutic strategy, because this outside one-third region of the meniscus is vascularized and could be self-healing. However, numerous meniscal damages occur in the inner two-thirds of the avascular zone where efforts to support self-healing can be challenging [3]. The current treatment strategies contain total or partial meniscectomy and meniscal allograft transplantation, however, each have their technical limitations in clinical practice. Cell-free based strategies bring new hope to restore injured menisci in situ to full functionality. Cell-free strategies aim to repair and regenerate injured tissue by recruiting endogenous stem/progenitor cells [4]. Moreover, scaffolds play a crucial role in cell-free techniques. Therefore, it is of great importance to fabricate a favorable scaffold for meniscus regeneration. This research aims to investigating the role of key element in meniscus scaffold fabrication and producing a biomimetic cell-free scaffold that could enhance meniscus defect in situ regeneration. Generally, both the biomechanical capacity and the biocompatibility played a critical role in meniscal scaffolds fabrication. On the one side, the delicate micro-structure similar to native meniscal collagen alignment could substantially determine the biomechanical capacity of the scaffold, on the other side, the suitable micro-environment close to the native meniscus extracellular matrix (MECM) could obviously enhance the biocompatibility of the scaffold. Hence, fabricating a cell-free scaffold with biomimetic micro-structure and micro-environment may be of great significance to the development of meniscus in situ regeneration.

3D printing technology combining with the biodegradable polymers has been used to design complicated scaffolds for various tissues or organs regeneration, such as bone, cartilage, heart and neural tissue [[5], [6], [7], [8]]. As to meniscus, both Chang Lee et al. and ZZ Zhang et al. used 3D printed polycaprolactone (PCL) scaffold to regenerate meniscus in animal model, and they all reported that those printed PCL scaffolds enhanced knee meniscus regeneration [[9], [10], [11]]. However, those printed PCL scaffolds did not mimic the native meniscus aligned micro-structure. In native meniscus, most collagen fiber bundles have a circumferential and radial orientation, and the aligned collagen micro-structure may be beneficial to withstanding forces such as shear, tension, and compression [12]. Yang yang et al. fabricated the radial and circumferential aligned scaffold using carbon nanotubes by electrically assisted nanocomposite 3D printing [13]. This scaffold displayed favorable biomechanical capacities, however, the biocompatibility of this scaffold was not assessed and was still unknown [14]. We attempted to achieve the intricate meniscal micro-structure using a 3D printing technology with well-designed program by PCL (widely used in Food and Drug Administration (FDA) approved devices) [15]. This aligned micro-structure may enhance the biomechanical capacity of the biomimetic meniscal scaffold and make it more conducive to withstand the complicated biomechanical requirement in vivo.

**Fig. 1:**
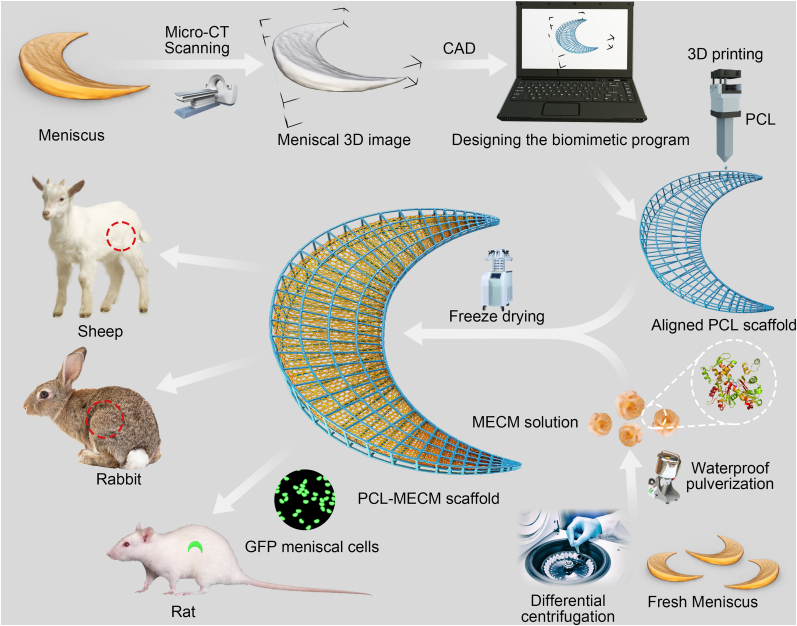
Schematic illustration of the whole study.

However, synthetic polymers present challenges in meniscus regeneration, such as the lack of bioactivity, hydrophobic properties, and degradation byproducts [12]. Recently, decellularized MECM was discovered to be beneficial to the amelioration of meniscal injuries. MECM could not only mimic the meniscus micro-environment but also preserve the tissue-specific biochemical composition of the native meniscal extracellular matrix (ECM), which can correctly regulate the behavior of endogenous stem/progenitor cells and enhance tissue regeneration [16,17]. MECM had been used as the bioactive factors to produce meniscal scaffolds for meniscus regeneration in rabbit model in our previous research, and they all demonstrated the favorable effects for repairing the injured meniscus [18,19]. Generally, the ECM derived scaffolds always displayed the lower biomechanical capacity in comparison with those from synthetic polymers derived scaffolds. On this basis, we attempted to combine the aligned PCL scaffolds with MECM to further produce cell-free PCL–MECM scaffold with biomimetic micro-structure and micro-environment. In this study, firstly, we produced the MECM and further evaluate the bioactive effects of MECM in the meniscal fibrochondrocytes (P3); then we fabricate the dually biomimetic PCL–MECM scaffolds and assess their biocompatibility; lastly, we further investigate the in situ regenerated evidence of the PCL–MECM scaffolds in both rabbit and sheep meniscectomy model (Fig. 1).

## 2 Materials and methods

### 2.1 Preparation and assessment of MECM

#### 2.1.1 Preparation of MECM

**Fig. 2:**
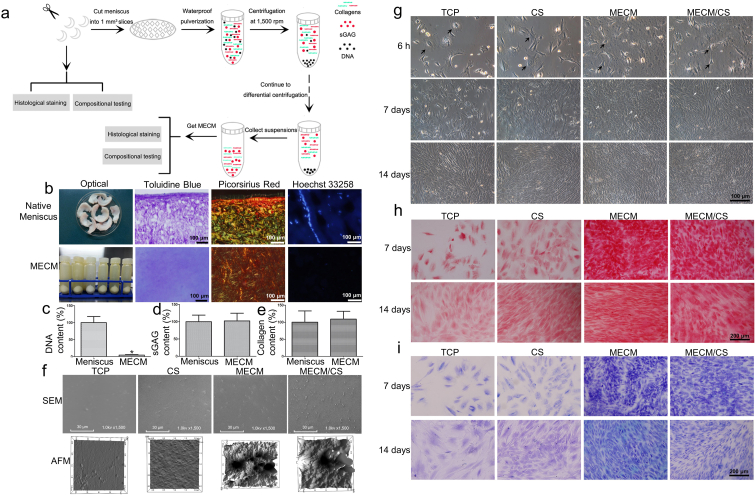
a) A schematic diagram showed the preparation of MECM using differential centrifugation approach. b), c), d), e) Comparison of the native meniscus and MECM. f) Topography of the various coated surfaces. g) Cellular appearance of meniscal fibrochondrocytes on various coated surfaces. h), i) Safranin O and toluidine blue staining of meniscal fibrochondrocytes after 7 and 14 days on various coated surfaces. All experiments were independently repeated in triplicate. Error bars represent standard deviation (*P < 0.05).

Porcine knee menisci (*n* = 30) were purchased from the abattoir immediately after slaughter, and transported to the laboratory on ice in sterile phosphate-buffered saline (PBS), pH 7.6. Menisci were cut into 1-mm^3^ slices under aseptic conditions. The slices were decellularized using a differential centrifugation approach. The slices were suspended and rinsed in sterile PBS three times, then homogenized using a tissue disintegrator to form a suspension slurry. The suspension of meniscal fragments was spun in a centrifuge (Beckman Allegra X-22R, USA) for 5 min at 1500 revolutions per minute (rpm) in an F0850 rotor. The pellet was removed and the suspension was re-centrifuged for 15 min at 2000 rpm. The resulting suspension was again centrifuged successively for another 20 min at 6000 rpm and 30 min at 10,000 rpm, MECM was collected from the 10,000 rpm pellet and intensively rinsed and centrifuged twice at 10,000 rpm in sterile PBS into a 3% (w/v) suspension. All centrifugation steps were performed under aseptic conditions. MECM was stored in sterile glassware at 4 °C for the following studies. A schematic diagram of the experiment is displayed in Fig. 2a.

#### 2.1.2 Comparison of the native meniscus and MECM

Fresh porcine meniscus was fixed in 4% PBS paraformaldehyde, dehydrated and embedded in paraffin, and cut in 10-μm thickness. MECM was cut by cryo-section (10-μm thickness), then fixed in acetone for 30 min at room temperature (RT) and washed with PBS. All specimens (*n* = 3 each group) were stained with toluidine blue and picrosirius red to identify the presence of proteoglycans and collagens. DNA from native meniscus and MECM was stained using Hoechst 33258 dye.

#### 2.1.3 Comparison and evaluation of biochemical components

1 mg dry weight porcine meniscus (*n* = 3 each group) and 1 mg dry weight MECM (*n* = 3 each group) were quantified as follows, respectively. The DNA content of both samples was determined by Hoechst 33258 approaches using the Quant-iT™ PicoGreen® dsDNA assay (Invitrogen, USA). The sulfated glycosaminoglycans (sGAG, a measure of content) were assessed using 1,9-dimethylmethylene blue solution by Tissue GAG Total Content DMMB Colorimetry Kit (GenMed Scientifics Inc., USA) [20]. The total collagen content was estimated by means of a conventional hydroxyproline assay using a Hydroxyproline Kit (Nanjing Jiancheng Bioengineering Institute, China) [21]. All procedures were performed according to the manufacturer's instructions.

#### 2.1.4 Preparation of biomimetic surfaces

Shark chondroitin sulfate (CS), was purchased from Sigma-Aldrich, and used as received without further purification. A surface modification procedure was performed as follows. Briefly, glass coverslips were immersed in strong acid overnight and rinsed three times with sterile PBS. After rinsing thoroughly with PBS, the coverslips were placed in six-well plates (Corning, USA). MECM, CS and MECM/CS solutions were prepared at a concentration of 1 mg/ml. MECM/CS solutions were produced by mixing MECM with CS in a 5:1 (w/w) ratio. The coverslip was incubated with 1 ml of various solutions per well, and the control group (TCP) surface was incubated with 1 ml of triple-distilled water per well and then air dried overnight at RT. All procedures were performed under aseptic conditions. Finally, the prepared six-well plates were sterilized by ^60^Co γ-irradiation (5 mRad) before use.

#### 2.1.5 Surface characterization of coated glass

The surface topography was observed by atomic force microscopy (AFM) (Asylum Research, USA) and scanning electron microscopy (SEM) (HITACHI/SU8020, Japan). All samples (*n* = 3 each group) were dehydrated through a series of graded alcohols and dried at RT. For AFM images, the dried samples were examined by MFP-3D AFM with a scan rate of 0.8 Hz. For SEM analysis, the dried samples were sputter-coated with gold–palladium and observed by SEM at 1 kV.

#### 2.1.6 Isolation and expansion of primary fibrochondrocytes from the rabbit meniscus

With approval from the local Ethics Committee of the Chinese PLA General Hospital, New Zealand White rabbits (*n* = 5, 3.0 kg) were euthanized, and tissues from inner two-thirds meniscus were obtained under aseptic conditions. The inner meniscus was diced into fragments <1 mm^3^. Then the meniscus fragments were digested in regular culture medium containing 0.2% collagenase type II (Sigma, USA), and transferred to an orbital shaker overnight at 37 °C. The medium formulation was as follows: Dulbecco's modified Eagle's medium (DMEM) (Sigma, USA), supplemented with 300 mg/ml l-glutamine, 50 mg/ml vitamin C, 100 U/ml penicillin and streptomycin (all Sigma, USA). After digestion, the mixture was washed twice with medium containing 10% fetal bovine serum (Gibco, USA) to remove excessive collagenase. Then 0.75 × 10^6^ cells were placed in tissue culture T-75 cm^2^ flasks (Corning, USA) at ~25% confluence in complete DMEM medium. After 1–2 weeks, cells in the T-75 flask approached 100% confluence and were trypsinized using 0.25% trypsin/EDTA (Gibco, USA), and then subcultured in T-75 flasks at a density of 1 × 10^4^ cells per cm^2^. The first subcultures were labeled as passage 1 (P1) cells, and so forth. This process was repeated until passage 4 (P4). For all studies, medium was replaced twice per week. A schematic diagram of the experiment is shown in Fig. S1.

#### 2.1.7 Culture rabbit meniscal fibrochondrocytes on coated surfaces

Confluent fibrochondrocytes (P3) were trypsinized, washed once, and suspended in culture medium. A 1-ml aliquot of cell suspension, which corresponded to approximately 3 × 10^4^ cells, was placed into each well. The plates were then incubated in a 5% CO_2_ humidified atmosphere at 37 °C for 30 min to allow cell adherence, and then 2-ml culture medium were added to each well. At 7 or 14 days, the cells were collected by centrifugation and snap-frozen in liquid nitrogen for further RNA extraction, as described in Fig. S1.

#### 2.1.8 Cell morphological examination

Cell morphology (*n* = 3 each group) was examined by phase-contrast microscopy and SEM. Phase-contrast microscopy was performed using an Olympus IX 70 inverted microscope (Olympus, Japan). For SEM, the cells/coated surface construct after 7 days culture was fixed in 2.5% glutaraldehyde, dehydrated in a graded ethanol series to 100% ethanol, treated with hexamethyldisilazane (HE). The construct was then sputter-coated with gold–palladium and observed by SEM at 5 kV (HITACHI/SU8020, Japan).

#### 2.1.9 Histological and immunohistochemical examination

The cells cultured in each well for 7 and 14 days were rinsed twice in PBS, and fixed in 4% PBS paraformaldehyde for 5 min. Samples (*n* = 3 each group) were then rinsed twice in PBS, dehydrated through a graded ethanol series, and stained with safranin O and toluidine blue for proteoglycans. For immunohistochemical assessment, the samples were immunolabeled with primary antibodies against collagen I (Abcam, UK), collagen II (Novus, USA), respectively, according to the manufactures’ instructions. Then secondary antibody of biotinylated goat antimouse and antirabbit IgG (Fuzhou Maixin Biotech, China) was applied for 30 min. Immunoactivity was detected by diaminobenzidine tetrahydrochloride (DAB). Hematoxylin served as a counterstain.

### 2.2 Preparation and assessment of the biomimetic cell-free PCL-MECM scaffolds

#### 2.2.1 Preparation of the cell-free PCL-MECM scaffolds

The anatomic shape of the medial meniscus of skeletally mature sheep was scanned by micro-CT and reconstructed by computer-aided design (Fig. S9). We then designed a printing program to mimic the native meniscal collagen circumferential and radial orientation. Micro-strands of 200 μm were fabricated for the sheep meniscus scaffold using PCL (Mn = 45000, Sigma-Aldrich, USA). The interconnecting micro-channels had a diameter of 750 μm or 1500 μm. PCL scaffolds were divided into four different types according to the alignment and micro-channel size: large pore (1500 μm)/aligned (LA), large pore (1500 μm)/unaligned (LU), small pore (750 μm)/aligned (SA), and small pore (750 μm)/unaligned (SU). The aligned structure means PCL scaffolds were printed according to the natural circumferentially and radially oriented collagen fiber arrangements within the meniscus.

The PCL scaffolds were then etched by alkaline solution to increase their hydrophilicity and surface area. PCL scaffolds were treated with 70% ethanol and subsequently etched with 5 M sodium hydroxide for 2 h [6]. After etching, the PCL scaffolds were washed in deionized H_2_O until the pH reached 7.4, and then air-dried. A MECM suspension (3% w/v) was then carefully poured onto the etched PCL scaffolds. Care was taken to ensure the pores were filled with MECM suspension, and scaffolds were incubated at −20 °C for 3 h. After complete freezing, the PCL-MECM scaffolds were lyophilized for 48 h under vacuum. The scaffolds were cross-linked under ultraviolet light (258 nm) for 4 h. The PCL-MECM scaffolds were then treated with 95% (v/v) alcohol containing 50 mM 1-ethyl-3-(3-dimethylaminopropyl) carbodiimide hydrochloride (EDAC) and 20 mM N-hydroxysuccinimide (Sigma-Aldrich, USA) for 24 h at 4 °C. Excessive EDAC was washed away by several washing steps with PBS. Then cell-free PCL-MECM scaffolds were washed in triple-distilled water to remove residual dioxane and again subjected to freeze drying. The scaffolds were finally sterilized by ^60^Co γ-irradiation (5 mRad) and stored at 4 °C prior to use.

#### 2.2.2 Sample characterization of cell-free PCL-MECM scaffolds

The gross examination of the cell-free PCL-MECM scaffolds (*n* = 3 each group) was performed by digital camera (Canon, Japan). The interior micro-structures of scaffolds were captured by SEM (HITACHI/SU8020, Japan). Biomechanical capacities, including the tensile modulus and compression modulus, on PCL-MECM scaffolds (*n* = 3 each group) were measured by a dedicated apparatus (Instron 5969, US). The cell–scaffold composite constructs (*n* = 3 each group) were observed by SEM (HITACHI/SU8020, Japan) after 3 days of culturing to assess their biocompatibility.

#### 2.2.3 Culture of rabbit meniscal fibrochondrocytes on PCL-MECM scaffolds and assessment of the scaffolds

A 50-μl aliquot of the cell suspension, which corresponded to approximately 5 × 10^4^ cells, was placed on PCL-MECM scaffolds (5 × 5 × 2 mm) in six-well plates. The cell viability (*n* = 3 each group) was assessed at 7 and 14 days using a cell Live/Dead assay kit, following the manufacturer's instructions, and examined by confocal microscopy. The images were analyzed with ImageJ software (National Institutes of Health, USA), and the cell viability rate was calculated as follows: (live cells/total cells) × 100%. The cellularity (*n* = 3 each group) was determined by DNA content after 7 and 14 days of culturing. sGAG production (*n* = 3 each group) was measured by the 1,9-dimethylmethylene blue (DMMB) approach. The collagen production (*n* = 3 each group) was measured by hydroxyproline content. sGAG content and collagen content were assessed in the cells/scaffolds, in medium and with only the scaffold. The sGAG content and collagen content secreted by cells were calculated as follows: cells/scaffolds + medium − scaffolds alone. After 7 and 14 days of culture, the cells/scaffolds were cryo-sectioned and stained with safranin O and toluidine blue. For immunofluorescence staining, the cryo-section samples (*n* = 3 each group) were stained with collagen type I and collagen type II, respectively. RNA was extracted from the cells and measured by RT-PCR following the manufacturer's instructions. The mRNA levels of dedifferentiated meniscal fibrochondrocytes (gene-specific primers, Table 1) in the various scaffolds (*n* = 3 each group) were in comparison with the P3 fibrochondrocytes before seeding. Each sample was assessed in triplicate.

**Table 1:** Primer sequences of target genes used for RT-PCR.

Target genes Primer sequences Direction GenBank accession number
Rbt Gapdh 5′-CAAGAAGGTGGTGAAGCAGG-3′ Forward NM_001082253.1
5′-CACTGTTGAAGTCGCAGGAG-3′ Reverse
Rbt Col1a2 5′-GCCACCTGCCAGTCTTTACA-3′ Forward NM_001195668.1
5′-CCATCATCACCATCTCTGCCT-3′ Reverse
Rbt Col2a1 5′-CACGCTCAAGTCCCTCAACA-3′ Forward XM_002723438.1
5′-TCTATCCAGTAGTCACCGCTCT-3′ Reverse
Rbt Sox-9 5′-GCGGAGGAAGTCGGTGAAGAAT-3′ Forward XM_002719499
5′-AAGATGGCGTTGGGCGAGAT-3′ Reverse
Rbt Col10a1 5′-CCACCAGGACAAGCAGTCAT-3′ Forward XM_002714724.1
5′-CACTAACAAGAGGCATCCCG-3′ Reverse
Rbt Aggrecan 5′-GGAGGAGCAGGAGTTTGTCAA-3′ Forward XM_002723376.1
5′-TGTCCATCCGACCAGCGAAA-3′ Reverse

#### 2.2.4 Culture of fibrochondrocytes from green fluorescent protein (GFP) rats on PCL-MECM scaffolds and in vivo assessment of the scaffolds

With approval from the local Ethics Committee of the Chinese PLA General Hospital, GFP rats (*n* = 5, 200–300 g) were euthanized, and tissue from the inner two-thirds of the menisci was obtained under aseptic conditions. Primary fibrochondrocytes from the GFP rats were isolated as described previously. A 50-μl aliquot of the GFP fibrochondrocyte suspension, which corresponded to approximately 5 × 10^4^ cells, was placed onto PCL-MECM scaffolds (5 × 5 × 2 mm) in six-well plates. The cell viability was assessed by confocal microscopy after 7 and 14 days of culture. The implantation experiment was performed with the committee guidelines for animal experiments at the Chinese PLA General Hospital. After culturing for 14 days, the cell–scaffold constructs were implanted subcutaneously in the dorsa of nude rats (*n* = 3 each group). The rats were sacrificed four weeks after surgery. The cell–scaffold constructs were harvested, fixed in 4% paraformaldehyde, dehydrated through a graded series of ethanol, embedded in paraffin, sectioned at a thickness of 10-μm, and assessed by HE, safranin O, toluidine blue, and immunofluorescence staining, the latter against type I collagen and type II collagen.

#### 2.2.5 In vivo degradation assessment of the scaffolds

With approval from the local Ethics Committee of the Chinese PLA General Hospital, Sprague−Dawley rats (200–300 g) were euthanized. The implantation experiment was performed with the committee guidelines for animal experiments at the Chinese PLA General Hospital. The various PCL-MECM scaffolds (5 × 5 × 2 mm) were implanted subcutaneously in the dorsa of rats (*n* = 3 each group), respectively. The rats were sacrificed at 1 week, 1 month and 2 months post-implantation. The scaffolds were harvested, fixed in 4% paraformaldehyde, dehydrated through a graded series of ethanol, embedded in paraffin, sectioned at a thickness of 10-μm, and assessed by HE staining.

### 2.3 The biomimetic cell-free PCL-MECM scaffolds implantation in rabbit model

#### 2.3.1 Surgical procedure in the rabbit model

This study was conducted under the committee guidelines for animal experiments at the Chinese PLA General Hospital. In total, 40 New Zealand white rabbits weighing 3.0 kg were randomly divided into the following four groups (*n* = 10 each group, Table 2) before operation: PCL-MECM scaffold, autograft, sham and control. The medial collateral ligament was cut to expose the posterior horn of the medial meniscus under anesthesia with intramuscular injections of 160 mg ketamine and 12 mg xylazine. A meniscectomy was then performed in both stifle joints of all rabbits. PCL-MECM scaffolds were implanted in the PCL-MECM scaffold group, and autologous menisci were implanted in the autograft group. Animals in the sham group only underwent exposure of the medial meniscus. Animals in the control group did not implant any tissues. The scaffolds and autologous menisci were sutured to the capsule at the level of the original meniscal rim, and the anterior and posterior horns were fixed to the meniscal ligaments. The capsule was then closed and the medial collateral ligament was reconstructed using resorbable sutures. After the operation, each rabbit received intramuscular penicillin injections to prevent knee joint infections, and each rabbit returned to its cage moving voluntarily. All rabbits were euthanized and assessed at 3 and 6 months post-surgery.

**Table 2:** Rabbits and sheep used for each group.

	PCL-MECM scaffold group	Autograft group	Sham group	Control group
Rabbits	10	10	10	10
Sheep	5	5	5	5

#### 2.3.2 X-ray imaging and magnetic resonance imaging (MRI)

X-ray imaging was performed in both the coronal and sagittal planes by a cabinet X-ray system (Faxitron X-ray, Hong Kong, China). MRI scans were made using a 7.0 T Bruker Biospec system (Bruker, Ettlingen, Germany). T2-weighted imaging (T2WI) was performed (repetition time = 3200 ms; echo time = 65 ms; number of slices = 15; slice thickness = 1 mm; number of replicate measurements = 3). Using Kellgren-Lawrence scores for knee X-ray imaging and using Whole-Organ Magnetic Resonance Imaging Scores (WORMS) for knee MRI imaging to assess the knee joint osteoarthritis, respectively [22,23].

#### 2.3.3 Macroscopic observations, histological and immunohistochemical analyses

The tibial plateau with the neo-menisci and femoral condyles (*n* = 3 each group) were evaluated and photographed (Canon, Japan). The tibail plateau coverage of the neo-menisci was calculated as follows: (the area of the neo-menisci/the area of the medial tibial plateau) × 100% [19]. The images were analyzed with ImageJ software (National Institutes of Health, USA). Then the neo-menisci were fixed in 4% paraformaldehyde for 3 days and embedded in paraffin. The corresponding distal femur and proximal tibia were decalcified in a 10% ethylene diamine tetraacetic acid (EDTA) solution for 28 days after being fixed in 4% paraformaldehyde for 3 days, dehydrated through a graded series of ethanol, and embedded in paraffin. The neo-menisci and bone–cartilage (*n* = 3 each group) were sectioned into 10-μm slices and stained with safranin O, toluidine blue, and immunohistochemical staining, the latter against type I collagen and type II collagen. Meniscal regeneration was assessed quantitatively by the Pauli score [24]. Cartilage degeneration of the femoral condyle and tibial plateau was evaluated using the Mankin score [25].

### 2.4 Statistical analysis

All quantitative data was presented as means ± standard deviation (SD). All statistical analyses were performed using SPSS v. 17.0. The MECM component and CCK-8 assay results were assessed by a Student's *t*-test, while the remaining statistical analyses were performed by one-way analysis of variance (ANOVA) followed by a post hoc Tukey test. *P*-values < 0.05 were considered to indicate significance for all statistical tests.

## 3 Results

### 3.1 Characterization and assessment of MECM

#### 3.1.1 Comparison of the native meniscus and MECM

Histological staining and quantification were performed to compare the biochemical composition of native meniscus with MECM. Fig. 2b demonstrates that toluidine blue and Picrosirius red staining of MECM were similar to native meniscus, which indicated that sGAG and collagens, respectively, were preserved in MECM. Hoechst 33258 positive staining revealed the presence of double-stranded DNA in normal meniscus, while DNA or DNA debris was absent in MECM.

The efficiency of the decellularization approach was evaluated by quantification analysis, which showed that this method could successfully remove cellular DNA and preserve meniscal ECM. Only 1.2 ± 0.15 ng DNA per mg tissue remained in MECM (Fig. 2c), corresponding to ~99% DNA reduction (P < 0.05). Both sGAG and collagen contents increased (Figs. 2d, 2e) by 3% and 9%, respectively, with MECM compared to native meniscus (P > 0.05).

#### 3.1.2 Surface characterization

The topography of the various biomimetic-coated surfaces was examined by AFM and SEM (Fig. 2f). The TCP surface was flat and smooth, whereas the CS surface was rougher than the TCP surface, with a root mean square (RMS) roughness factor of 1.3 nm. The MECM surface and MECM/CS surface were more uneven and rougher than the other two surfaces, and the RMS roughness of MECM/CS surface increased from 19.1 to 25.6 nm after addition of CS.

#### 3.1.3 Dedifferentiated meniscal fibrochondrocytes cultured on the coated surfaces

##### 3.1.3.1 Cell morphology

The meniscal fibrochondrocytes had varying morphologies on different coated surfaces, as visualized by phase-contrast microscopy (Fig. 2g) and SEM (Fig. S4). Most cells presented a “round” morphology on the TCP surface at 6 h post-seeding and had a polygonal morphology on the CS surface. In contrast, cells on the MECM and MECM/CS surfaces showed a contracted cytoplasm, while cells on MECM/CS surface contracted more deeply than MECM surface (Fig. S5). After 7 days in culture, all cells showed an elongated fibroblast-like morphology on TCP and CS coated surfaces, whereas some cells on the MECM and MECM/CS surfaces had an identical appearance to primary fibrochondrocytes. The cells on the various coated surfaces reached different levels of confluence; cells grown on the MECM and MECM/CS surfaces attained 90% confluence, compared to 40% and 50% confluence, respectively, for cells grown on the TCP and CS surfaces. SEM assessment showed that cells on MECM and MECM/CS-coated surfaces had a rougher morphology compared to those on the other two surfaces (Fig. S4), which indicated secretion of a greater quantity of ECM. After 14 days in culture, cells grown on TCP and CS surfaces achieved confluence, whereas cells on the other two surfaces were over-confluent.

##### 3.1.3.2 Histology and immunohistochemistry

Histology and immunostaining were performed to estimate the density and distribution of the specific matrix on the various coated surfaces (Figs. 2h, 2i, S7). At day 7 and 14, sGAG and collagen II were distributed intensively on both the MECM and MECM/CS surfaces, which also presented a greater number of cells compared to the TCP surface and CS surface. Whereas collagen I was distributed weakly throughout the various coated surfaces. At day 7 and 14, there was no significantly different staining for collagen I on the various coated surfaces.

### 3.2 Characterization and assessment of the biomimetic cell-free PCL-MECM scaffolds

#### 3.2.1 SEM assessment

**Fig. 3:**
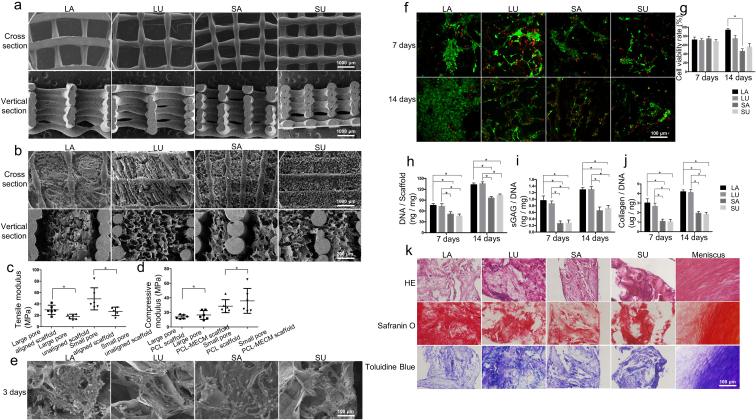
a), b) SEM assessment of PCL and PCL-MECM scaffolds in the cross-sectional view and vertical section views. c), d) Biomechanical assessment of the various scaffolds. e) SEM assessment of meniscal fibrochondrocytes after 3 days on various PCL-MECM scaffolds. f), g) Confocal microscopy image and cell viability rate of the meniscal fibrochondrocytes after 7 and 14 days on various PCL-MECM scaffolds. h), i), j) DNA content and ECM production of meniscal fibrochondrocytes after 7 and 14 days on various PCL-MECM scaffolds. k). HE, safranin O, and toluidine blue staining of the rat GFP meniscal fibrochondrocytes and PCL-MECM scaffolds constructs in nude rats 4 weeks after subcutaneous implantation. All experiments were independently repeated in triplicate. Error bars represent standard deviation (*P < 0.05).

The biomimetic cell-free scaffold, which mimics the anatomical shape of the meniscus and has a similar micro-structure, was printed using PCL (Fig. S10). The PCL scaffold was treated with an alkaline solution to improve its hydrophilicity [6]. To further investigate the effect of the pore size and aligned structure on the scaffold, different scaffolds were produced: large pore (1500 μm)/aligned scaffold (LA), large pore (1500 μm)/unaligned scaffold (LU), small pore (750 μm)/aligned scaffold (SA), small pore (750 μm)/unaligned scaffold (SU). SEM imaging of the cross-section showed a circumferential and radial orientation of the PCL structure in the aligned scaffolds, and only a horizontal or vertical cross-point architecture in the unaligned scaffolds (Fig. 3a). Some of the PCL macroporous structure and MECM microporous structure existed in all PCL-MECM scaffolds, and the MECM microporous structure was observed in the PCL macroporous structure (Fig. 3b). SEM imaging of the vertical section showed that the PCL scaffold mainly formed a vertically-aligned structure, and MECM filled the empty spaces and further formed some of a microporous structure.

#### 3.2.2 Biomechanical assessment

Biomechanical tests showed that the aligned PCL-MECM scaffolds possessed a better tensile modulus than the unaligned scaffolds (Fig. 3c). The biomechanical assessment showed that the scaffolds with large pores possessed a lower compressive modulus than scaffolds with small pores (Fig. 3d).

#### 3.2.3 Biocompatibility and viability assessment

Meniscal fibrochondrocytes were seeded on the different PCL-MECM scaffolds and cultured for 3 days. SEM imaging results showed good cell adhesion and high ECM secretion for all four scaffolds, indicating the PCL-MECM scaffolds displayed good biocompatibility (Fig. 3e). The passaged meniscus fibrochondrocytes were cultured on the different PCL-MECM scaffolds for 14 days and evaluated viability. The fibrochondrocyte viability on the large pore scaffolds was better than that on the small pore scaffolds (Fig. 3f, g).

#### 3.2.4 Chondrogenic differentiation assessment

The passaged meniscus fibrochondrocytes were cultured on the different PCL-MECM scaffolds for 14 days and evaluated the chondrogenic differentiation in vitro. The increased DNA content of fibrochondrocytes on all scaffolds suggest that PCL-MECM scaffolds enhances cell growth (Fig. 3h). Collagen type II and Sox-9 mRNA expression was highly upregulated in cells seeded on large pore scaffolds compared to cells seeded on small pore scaffolds (Fig. S11). The upregulation of collagen type II mRNA was in accordance with our immunofluorescence results (Fig. S12), which showed a significant increase in total collagen content after 7 and 14 days of culturing on large pore scaffolds (Fig. 3j). Similarly, the sGAG/DNA content of cells cultured on large pore scaffolds was greater than that on small pore scaffolds after 7 and 14 days of culturing (Fig. 3i), which was verified by safranin O and toluidine blue staining (Fig. S13).

Green fluorescent protein (GFP) rat meniscus-derived fibrochondrocytes were seeded on PCL-MECM scaffolds and cultured for 14 days (Fig. S14). We then implanted the cell–scaffold composites into the dorsal subcutaneous tissue of nude rats for 28 days to trace the chondrogenic differentiation capacity of the PCL-MECM scaffold in vivo. GFP rat meniscus-derived fibrochondrocytes were still alive 28 days post-implantation, as demonstrated by positive GFP staining (Figs. S15 and S16). Additionally, the PCL-MECM scaffold could induce the GFP fibrochondrocytes to differentiate in vivo, as demonstrated by the positive staining of neo-menisci with safranin O and toluidine blue (Fig. 3k). Immunofluorescence staining of collagen type I and type II around the GFP cells further verified the cell–scaffold composites could form meniscus-like tissue in vivo (Figs. S15 and S16).

#### 3.2.5 In vivo degradation assessment

Both the PCL (White void spaces) and MECM (Black arrows indicate) could be observed in the scaffolds after 1 week implantation (Fig. S17). There were still some residual PCL, while few MECM in the scaffolds after 1 month implantation. It could only observe degraded PCL in the scaffolds after 2 months implantation. Therefore, the MECM may degrade completely after 1 month implantation, and the PCL will not degrade completely after 2 months implantation.

### 3.3 The biomimetic cell-free PCL-MECM scaffolds promoted meniscus in situ regeneration in vivo

#### 3.3.1 The biomimetic cell-free PCL-MECM scaffolds promoted meniscus in situ regeneration in rabbit model post 3 and 6 months implantation

**Fig. 4:**
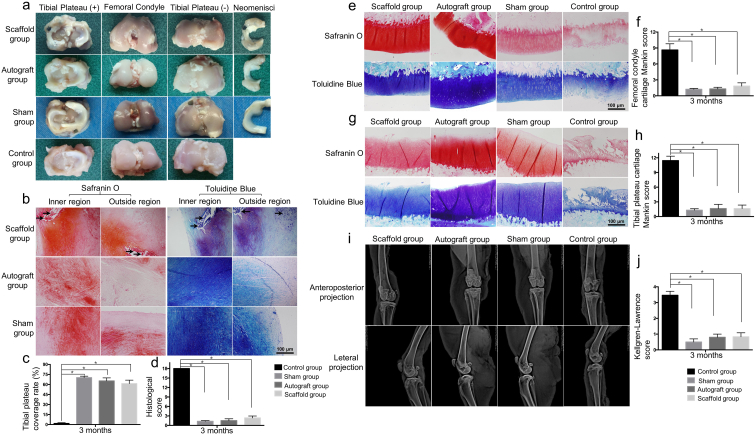
Comprehensive assessment of the neo-menisci in a rabbit meniscus injury repair model 3 months post-surgery. a) Macroscopic analysis of neo-menisci and articular cartilage of the corresponding tibial plateau and femoral condyles. b) Histological staining of the neo-menisci (Black arrows indicate degraded PCL). c) Tibial plateau coverage rate of neo-menisci. d) Histological scores of the neo-menisci. e), f), g) h) Histological staining and scores of articular cartilage in the corresponding tibial plateau and femoral condyles, respectively. i), j) X-ray assessment and scores of the corresponding knee joints. All experiments were independently repeated in triplicate. Error bars represent standard deviation (*P < 0.05).

**Fig. 5:**
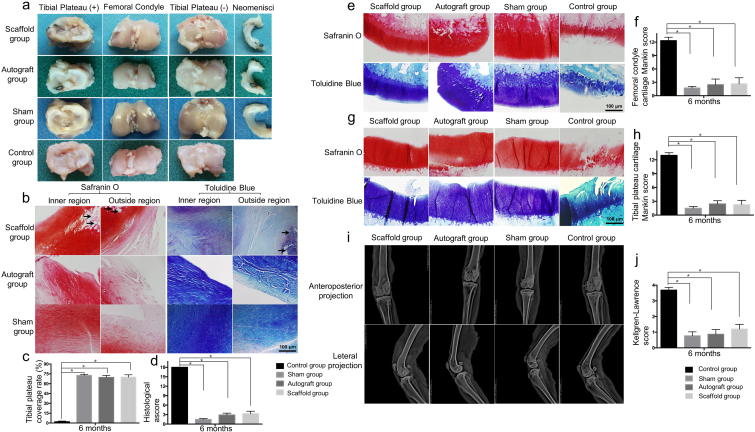
Comprehensive assessment of the neo-menisci in a rabbit meniscus injury repair model 6 months post-surgery. a) Macroscopic analysis of neo-menisci and articular cartilage of the corresponding tibial plateau and femoral condyles. b) Histological staining of the neo-menisci (Black arrows indicate degraded PCL). c) Tibial plateau coverage rate of neo-menisci. d) Histological scores of the neo-menisci. e), f), g) h) Histological staining and scores of articular cartilage in the corresponding tibial plateau and femoral condyles, respectively. i), j) X-ray assessment and scores of the corresponding knee joints. All experiments were independently repeated in triplicate. Error bars represent standard deviation (*P < 0.05).

The large pore, aligned PCL-MECM scaffold was the optimal scaffold to repair defective rabbit medial menisci based on the biomechanical test results, viability, and chondrogenic differentiation (Table 3). To assess their effect on meniscus regeneration, we implanted the autograft menisci in rabbits that had undergone total meniscectomy. In the PCL-MECM scaffold group, some neo-menisci were observed and well covered the corresponding tibial plateau cartilage, which resembled anatomical characteristics of the meniscus autograft group and sham group, 3 and 6 months post-implantation (Fig. 4a and c; 5a, 5c). Whereas there is no obvious neo-meniscus formation in control group. Additionally, in terms of cartilage protection, there was little obvious damage to the articular cartilage surface in the PCL-MECM scaffold group and autograft group, in contrast mild to moderate wear in the control group.

**Table 3:** Comparison of the various cell-free PCL-MECM scaffolds.

	LA group	LU group	SA group	SU group
Tensile modulus	+	–	+	–
Compressive modulus	+	+	–	–
Cell viability	+	–	–	–
Chondrogenic differentiation in vitro	+	+	–	–
Chondrogenic differentiation in vivo	+	+	+	+

+ good, - normal.

Histological staining confirmed that the neo-menisci in the PCL-MECM scaffold group showed meniscus-like heterogeneous staining characteristics (Figs. 4b, 5b, S18). In the PCL-MECM scaffold group, safranin O, toluidine blue and collagen type II staining in the inner region and collagen type I staining in the outer region were both positive at 3 and 6 months post-implantation, and the staining intensity was increasingly stronger from 3 months to 6 months post-implantation. Specifically, on the one side, there were plentiful spindle-shaped fibroblast-like cells and few round-shaped chondrocyte-like cells in the inner region of the neo-menisci at 3 months post-implantation, while there were increasingly more and more round-shaped chondrocyte-like cells in the inner region of the neo-menisci at 6 months post-implantation. On the other side, the outer region of the neo-menisci displayed numerous spindle-shaped fibroblast-like cells at 3 months post-implantation, while there were populated by abundant elongated fibroblast-like cells in the outer region of the neo-menisci at 6 months post-implantation. Meanwhile, the cellular numbers of the neo-menisci in both inner and outer regions increased strikingly from 3 months to 6 months post-implantation. Notably, the collagen fiber alignment changed from unoriented structure at 3 months post-implantation to oriented formation at 6 months post-implantation. Additionally, the histological score in the PCL-MECM scaffold group was significantly better than that in the meniscectomy group, and it was similar to that in the meniscus autograft and sham groups (Fig. 4d; 5d). Lastly, there were obviously some degraded PCL in both the inner region and the outer region of the neo-menisci, while no obvious MECM in the neo-menisci at both 3 months and 6 months post-implantation.

The chondroprotective effect of neo-meniscus was assessed by histological examination and Mankin scores. Histological examination revealed that the articular cartilage surface was better preserved in the PCL-MECM scaffold group than that in the control group over 6 months, which similar to the macroscopic assessment. Meanwhile, Mankin scores in the PCL-MECM scaffold group were significantly better than those of the control group at 3 and 6 months post-implantation (Fig. 4e, f, 4g, 4h; 5e, 5f, 5g, 5h).

#### 3.3.2 The X-ray and MRI assessment after PCL-MECM scaffolds implantation in rabbit model post 3 and 6 months

The degeneration of rabbit knee joints was assessed by X-ray. The knee X-ray examination revealed obvious narrowing of the knee joint space and osteophyte formation in the control group. Similarly, the Kellgren-Lawrence scores in control group were higher than those in the other three groups. In contrast, there was no obvious articular cartilage degeneration in the PCL-MECM scaffold group (Fig. 4i, j; 5i, 5j), which similarly to the autograft group and sham group.

Knee MRI was also used to assess meniscus regeneration and cartilage degeneration. On the one hand, MRI examination displayed obvious synovial inflammation signals and bone marrow edema in the control group, whereas no obvious inflammatory signals in the PCL-MECM scaffold group. On the other hand, there were distinct signals indicating the neo-meniscus in the PCL-MECM scaffold group, whereas only synovial inflammatory signals were observed in the control groups. Notably, the neo-meniscus images were demonstrated increasingly clearly in the PCL-MECM scaffold group from 3 months to 6 months post-implantation. Moreover, the knee MRI results showed lower WORMS scores in the PCL-MECM scaffold group than that in the control group at 3 and 6 months post-implantation (Fig. S19).

## 4 Discussion

The primary objective of this study was to design a functional meniscus scaffold which could apply to clinical practice based on the cell-free strategies. Meniscal cell-free implants such as the Collagen Meniscus Implant (CMI®) and Actifit® scaffolds are available for the treatment of meniscal injuries in clinic currently. However, recent studies have displayed that the current meniscus scaffolds did not display satisfactory results, especially their long-term effectiveness could be an issue, none have displayed chondroprotective effect in humans [26]. We have combined the cell-free strategies with ECM-based technique and 3D printing approach to develop a biomimetic PCL-MECM scaffold to meniscus in situ regeneration. In order to improving effectiveness, it is reasonable for combination of both biological derived materials and synthetic polymers together to enhance both biocompatibility and biomechanical capacity of meniscus scaffold. Cell-free scaffold is a one-step clinical approach for tissue in situ regeneration with little side-effects in comparison with cell-based strategies, which always need recruit endogenous stem/progenitor cells [27]. In terms of cell-based strategies, it always need two surgical interventions when using autologous cells. Meanwhile, as for using allogeneic cells, immunogenicity, ethic and cell source will be a big obstacle [28]. Currently, we have provided the positive evidence that the biomimetic cell-free PCL-MECM scaffold could not only correctly orchestrate the meniscus in situ regeneration, but also possess the obviously chondroprotective effect in both rabbit and sheep meniscectomy model. Therefore, the cell-free PCL-MECM scaffold may indicate a highly clinical relevant therapeutic option for in future meniscus regeneration.

In first part, we have produced the MECM by waterproof pulverization and differential centrifugation approach. The biochemical analysis showed that MECM could preserve the main native meniscal ECM components, including sulfated glycosaminoglycans and collagen, yet cellular DNA was effectively removed [12]. Both two kinds of meniscus ECM play a critical role in meniscus regeneration. To further investigate the biocompatible effect of MECM on passaged meniscal cells, glass coverslips were coated with (i) chondroitin sulfate (CS), (ii) MECM, or (iii) MECM/CS (ratio of 5:1), which were compared to a TCP control group. The MECM surface showed a rough appearance, which increased with CS (MECM/CS). We speculate that an uneven and rough surface increases the contact area for cell–matrix interactions and mimics the three-dimensional in vivo micro-environment. The MECM surface, followed by the MECM/CS surface, showed the highest adherence rate at 6 h post-seeding. The increased attachment was likely due to the collagen in MECM, since collagen promotes cell adhesion [29,30]. The CS surface showed the lowest attachment rate [31]. This suggests that MECM facilitates cell growth and maintains the viability by mimicking the in vivo micro-environment of the meniscal cells. CCK-8 assays further confirmed that MECM enhanced the proliferation of passaged cells.

SEM assessment presented that cells on MECM and MECM/CS surface were surrounded by dense ECM after 7 days culture. The collagen type II mRNA expression was upregulated in cells passaged on MECM or MECM/CS. Passaged cells may redifferentiate when cultured on MECM and MECM/CS, because collagen type II plays a critical role in maintaining the meniscal structure and is closely related to osteoarthritic prevention [32,33]. The upregulation of collagen type II expression was in accordance with the immunohistochemical results, and a significant increase in the total collagen content was observed after 7 and 14 days of culturing on the MECM or MECM/CS surfaces. Similarly, the sGAG/DNA content of cells cultured on the MECM and MECM/CS surfaces, as verified by safranin O and toluidine blue staining, was greater than that of cells cultured on the TCP and CS surfaces for 7 and 14 days. However, based on the collagen type II expression, fibrochondrocytes on MECM/CS showed less redifferentiation capacity than those on MECM. Some studies have reported that GAGs, including CS may decrease the expression of collagen II, which may be due to the excessive hydration of GAGs that increases the distance between cells and the surrounding MECM, thereby decreasing cell–matrix interactions [[34], [35], [36]]. In conclusion, MECM could guide the chondrogenesis of fibrochondrocytes and serve as the bioactive factors for meniscal regeneration in vitro.

In the second part, sheep meniscus was scanned by micro-CT and reconstructed subsequently, then printed by a well–designed program that mimics the circumferential and radial arrangement of native meniscal collagen fibers. The PCL scaffold was treated with an alkaline solution to improve its hydrophilicity. Then MECM was loaded into the hydrophilic PCL scaffold, and the biomimetic micro-structure and micro-environment cell-free PCL–MECM scaffold was produced by freeze–drying. In the cross-section views showed a circumferential and radial orientation structure which mimic native meniscus collagen alignment, while in the vertical section views showed a vertically-aligned structure in the aligned PCL scaffolds. In all cases, the MECM microporous structure was observed in the PCL macroporous structure. There is no doubt that the MECM microporous structure will provide a well microenvironment for the endogenous stem cell repairing and regeneration. Moreover, the aligned PCL structure will enhance the biomechanical capacity of the PCL-MECM scaffolds. In fact, on the one hand, the aligned PCL-MECM scaffolds displayed better biomechanical characteristics than the unaligned PCL-MECM scaffolds. Because the tensile modulus for both the aligned and unaligned scaffolds were all less than in the human native meniscus (105 ± 58 MPa) [37]. The higher tensile modulus of all the PCL-MECM scaffolds may be close to that in the native meniscus. On the other hand, the large pore PCL-MECM scaffolds displayed better biomechanical characteristics than the small pore PCL-MECM scaffolds. Because the compressive modulus all in the PCL-MECM scaffolds was greater than that in the human native meniscus (1.52 ± 0.59 MPa) [38]. The lower compressive modulus of the scaffolds may be close to that in the native meniscus.

The fibrochondrocyte viability on the large pore scaffolds was better than that on the small pore scaffolds. The increased DNA content of fibrochondrocytes on all scaffolds suggest that the MECM enhances cell growth by mimicking the meniscal micro-environment. Collagen type II and Sox-9 mRNA expression was highly upregulated in cells seeded on large pore scaffolds compared to cells seeded on small pore scaffolds. The upregulation of collagen type II mRNA was in accordance with our immunofluorescence results, which showed a significant increase in total collagen content after 7 and 14 days of culturing on large pore scaffolds. Similarly, the sGAG/DNA content of cells cultured on large pore scaffolds was greater than that on small pore scaffolds after 7 and 14 days of culturing, which was verified by safranin O and toluidine blue staining. These results convincingly demonstrate that the large pore scaffold could enhance the proliferative ability, viability, and redifferentiation capacity of cultured fibrochondrocytes, which may be due to the higher MECM content and biocompatibility of the large pore scaffolds compared to the small pore scaffolds. GFP rat meniscus-derived fibrochondrocytes could still be alive 28 days post-implantation, moreover, the PCL-MECM scaffold could induce the GFP fibrochondrocytes to the chondrogenic differentiate in vivo, as demonstrated by the positive collagen type I and type II staining of neo-menisci. The PCL-MECM scaffold in vivo degradation assessment had displayed that MECM will degrade completely after 1 month implantation, while PCL will last more than 2 months. It was showed that 28 days is enough for cellular chondrogenic differentiate by the GFP rat fibrochondrocytes ectopic chondrogenic differentiation result. Hence it is suggested that the PCL-MECM scaffold could provide enough differentiation time for cells performing chondrogenesis in vivo before MECM completely degradation. Meanwhile, the residual PCL scaffold could continue to act as a biomechanical supporter to fulfill the complicated biomechanical requirement in vivo for a long time due to the PCL slow degradation rate.

In the third part, the therapeutic objectives of cell-free PCL-MECM scaffold after meniscal injury in an animal model are to regenerate functional neo-meniscus in situ and to protect the knee joint from osteoarthritis development. At the beginning, the rabbit has been chosen as the translational animal model due to their small size and the lower cost [39]. In the cell-free PCL-MECM scaffold group, a significant neo-menisci were observed and well covered the corresponding tibial plateau cartilage, which resembled anatomical characteristics of the meniscus autograft group, 3 and 6 months post-implantation. While, in the control group, there is no tissue regeneration which may demonstrate the differences in endogenous repairing capacity between PCL-MECM scaffold group and control group in rabbit model. Histological staining confirmed that the neo-menisci showed meniscus-like heterogeneous staining. On the one hand, the small MECM pore in PCL-MECM scaffold may contribute to the migration of endogenous cells and correct orchestration of the new tissue regeneration and remodeling. On the other hand, the degraded PCL in the neo-menisci further convinced our speculation that the residual PCL scaffolds may still provide a biomechanical supporter for meniscus in situ regeneration at both 3 months and 6 months post-implantation. Meanwhile, the histological score in the PCL-MECM scaffold group was significantly better than that in the meniscectomy group, and it was similar to that in the meniscus autograft and sham groups. The histological examination and Mankin scores showed that the PCL-MECM scaffold could protect the tibial plateau and femoral condylar articular cartilage well, and the knee X-ray examination revealed obvious articular cartilage degeneration and higher Kellgren-Lawrence scores in the meniscectomy group, whereas there was no obvious narrowing of the knee joint space or osteophyte formation in the PCL-MECM scaffold group. The knee MRI results showed some neo-menisci and lower WORMS scores in the PCL-MECM scaffold group 3 and 6 months post-implantation.

Based on the rabbit repairing results, we further verified the safety and effectiveness of the cell-free PCL-MECM scaffold in sheep partial medial menisci defects model (Fig. S19, S21, S22, S23). On the one hand, the sheep have a knee load bearing pattern more similar to that of humans than rabbits [40], on the other hand, sheep meniscus has similar structure to humans. In terms of the anatomical match aspect, the PCL-MECM scaffold could be easily fit the meniscus defect through 3D printing approach by the reconstructed meniscus image via Micro-CT. Meanwhile this advantage could facilitate the PCL-MECM scaffold accurate match in practical injured joints and be beneficial to clinically surgical operation. Neo-menisci were observed in the PCL-MECM scaffold group and well covered the corresponding tibial plateau cartilage 3 months post-operation. Histological staining verified that the neo-menisci displayed heterogeneous meniscus-like staining, and the histological score in the PCL-MECM scaffold group was significantly greater than that in the meniscectomy group and similar to that in the meniscus autograft group and sham group. Similarly, the degraded PCL in the neo-menisci also suggested the residual PCL scaffold may still provide a biomechanical supporter for meniscus in situ regeneration at 3 months post-implantation. The histological examination and Mankin scores showed that the PCL-MECM scaffold could protect the tibial plateau and femoral condylar articular cartilage well. Additionally, knee X-ray examination displayed obvious articular cartilage degeneration and higher Kellgren-Lawrence scores in the meniscectomy group, whereas there was no obvious knee joint space narrowing or osteophyte formation in the PCL-MECM scaffold group. The MRI image results showed some neo-menisci and lower WORMS scores in the PCL-MECM scaffold group 3 months post-operation.

**Fig. 6:**
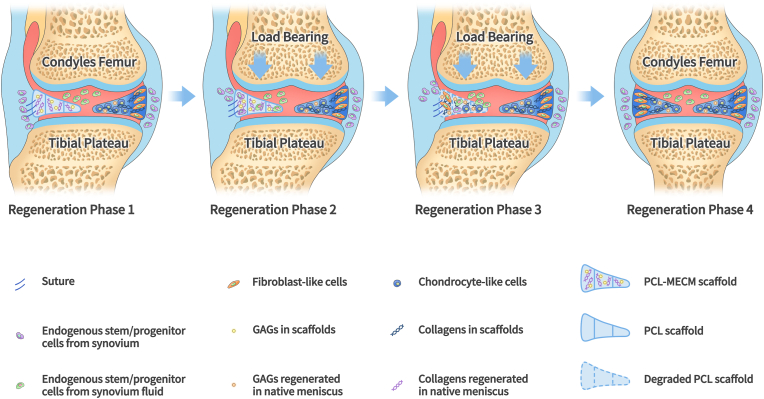
Schematic illustration of the degradation process of the PCL-MECM scaffold and the regeneration process of neo-meniscus.

Based on both the rabbit and the sheep meniscus repairing model, it could draw the conclusion that the PCL-MECM scaffold may achieve a certain balance between scaffold degradation and meniscus regeneration. It means that the cell-free PCL-MECM scaffold degradation rate may match neo-menisci in situ regeneration rate in vivo to some extent. PCL-MECM scaffold may play two significant roles in meniscus regeneration: on the one side, acting as the bioactive factors to regulate endogenous stem/progenitor cells behavior and formation to neo-menisci at the previously 1 month before MECM completely degradation; on the other side, acting as a favorable biomechanical supporter to fulfill the complicated biomechanical requirement in vivo due to the greatly slow degradation rate of PCL scaffold. A schematic diagram may demonstrate the degradation process of PCL-MECM scaffold and the regeneration process of neo-meniscus (Fig. 6).

There are still some limitations in this study. Despite chondrogenic differentiation of the PCL-MECM scaffold has been convinced, recent study displayed that the meniscogenic differentiation is also important to meniscus regeneration [41]. Future studies will further investigate the meniscogenic differentiation of the PCL-MECM scaffold, such as Mohawk. Meanwhile, we also need to figure out the effect of PCL-MECM scaffold on the human meniscal cells, including cellular differentiation and viability. Moreover, future work will further focus on the distinguishing the detailed role between MECM and PCL in meniscus regeneration. On the one side, identify how does the MECM affect the migration, proliferation and differentiation on endogenous stem/progenitor cells; on the other side, study the PCL degradation characteristic and their biomechanical supporter role in meniscal regeneration. Last but not least, it is also necessary to confirm the knee joint protective effects of the cell-free PCL-MECM scaffold within an extended observation time on injured sheep menisci model.

In conclusion, the dually biomimetic cell-free PCL-MECM scaffold could well mimic the micro-structure and micro-environment of native meniscus, and displayed superior biocompatibility and strong biomechanical capacities. Moreover, cell-free PCL-MECM scaffold could achieve good in situ meniscus regeneration, protect the knee joint articular cartilage, and postpone the development of knee osteoarthritis. PCL-MECM scaffold may represent a promising approach worthy of further investigation in preclinical applications.

## Additional information

The online version of this article contains supplementary material, which is available to authorized users.

## CRediT authorship contribution statement

**Weimin Guo:** Writing – original draft, Investigation, Data curation, Formal analysis, Visualization. **Mingxue Chen:** Writing – original draft, Investigation, Data curation. **Zhenyong Wang:** Investigation, Data curation, Formal analysis. **Yue Tian:** Data curation, Formal analysis. **Jinxuan Zheng:** Data curation. **Shuang Gao:** Formal analysis. **Yangyang Li:** Methodology. **Yufeng Zheng:** Conceptualization, Supervision. **Xu Li:** Software. **Jingxiang Huang:** Methodology. **Wei Niu:** Investigation, Methodology. **Shuangpeng Jiang:** Investigation, Methodology. **Chunxiang Hao:** Investigation, Methodology. **Zhiguo Yuan:** Methodology. **Yu Zhang:** Methodology. **Mingjie Wang:** Methodology. **Zehao Wang:** Methodology. **Jiang Peng:** Project administration. **Aiyuan Wang:** Project administration. **Yu Wang:** Project administration. **Xiang Sui:** Resources. **Wenjing Xu:** Project administration. **Libo Hao:** Project administration. **Xifu Zheng:** Supervision. **Shuyun Liu:** Supervision. **Quanyi Guo:** Conceptualization, Supervision.

## Declaration of competing interest

The authors declare no conflict of interest.
