# Supplementary material for: 3D-printed cell-free PCL–MECM scaffold with biomimetic micro-structure and micro-environment to enhance in situ meniscus regeneration
Source: Bioact Mater. 2021 Mar 27;6(10):3620–33. doi: 10.1016/j.bioactmat.2021.02.019 (PMC8039774; doi:10.1016/j.bioactmat.2021.02.019)
Supplement: Multimedia component 1 [file mmc1.docx]

**Supplementary data**

**1. Materials and Methods**

**1.1. Preparation and assessment of MECM**

**1.1.1. Cell viability assessment**

Cell viability was assessed at 7 and 14 days (*n* = 3 each group) culture using a cell Live/Dead assay kit (Sigma, USA) according to the manufacturer’s instructions. Briefly, the construct was washed twice in PBS, and incubated in 5 × 10^−3^ mg/ fluorescein diacetate (FDA) 5 min at RT in the dark. Then, the FDA was aspirated and again washed twice in PBS. The construct was incubated in another 5 × 10^−3^ mg/ml propidium iodide (PI) for 5 min at RT in the dark. PI was removed, and the construct was washed twice in PBS and examined by confocal microscopy (Olympus IX 81, Japan). The images were analyzed with ImageJ software (National Institutes of Health, USA), and the cell viability rate was calculated as follows: (live cells / total cells) × 100%.

**1.1.2. Cell proliferation assay**

Cell counting kit-8 (Dojindo CCK-8 kit, Tokyo, Japan) was used to quantitatively evaluate the cell proliferation of MECM. The 96-well plates were incubated with 62-μl 1 mg/ml MECM per well in MECM group, while TCP was incubated with 62-μl triple-distilled water per well, then air dried overnight at RT. All coated procedures were performed under aseptic conditions. A fibrochondrocyte (P3) suspension (200 μl), which corresponded to approximately 2.5 × 10^3^ cells, was placed into each well. The plates were then incubated in a 5% CO_2_ humidified atmosphere at 37°C to allow for cell growth. At 1, 3, 5, and 7 days, 20-μl CCK-8 solution were added to each well, followed by incubation at 37 for 2 h. The optical density (OD) at 450 nm was determined using a microplate reader (EPOCH TAKE 3, Bio Tek, USA). Six replicates were performed per sample.

**1.1.3. Determination of adhesion and cellularity**

Cell adhesion was evaluated by determining DNA content. Six hours after cell seeding (3 x 10^4^ cells per coverslip), meniscal fibrochondrocytes in each cells/coated surface construct (*n* = 3 each group) were rinsed in PBS, trypsinized and collected by centrifugation. Total DNA content for each sample was determined by the Quant-iT™ PicoGreen^®^ dsDNA assay (Invitrogen, USA), according to the manufacturer’s instructions. Cellularity was determined by DNA content after 7 and 14 days of culture in the same way. Lastly, DNA content was transformed to cell number using a conversion factor of 7.7 pg DNA/cell [[1](#_ENREF_1)].

**1.1.4. Determination of sGAG and collagen production**

The fibrochondrocytes in each well (*n* = 3 each group) were scraped from the bottom using a cell scraper. sGAG production was measured by 1,9-dimethylmethylene blue (DMMB) approach, using Cell GAG Total Content DMMB Colorimetry Kit (GenMed Scientifics Inc., USA). Collagen production was measured by hydroxyproline content, followed by conversion to collagen content, using a Hydroxyproline Kit (Nanjing Jiancheng Bioengineering Institute, China). Both biochemical analyses were performed according to the manufacturer’s instructions.

**1.1.5. Gene expression analysis using real-time quantitative reverse transcriptase polymerase chain reaction (RT-qPCR)**

RNA was extracted from samples of ground tissue and passaged fibrochondrocytes grown on the various surfaces (*n* = 3 each group) by adding TRIzol (Life Technologies, USA) or using the RNeasy kit (Qiagen, Germany) according to the manufacturer’s instructions. RNA was reverse transcribed to cDNA using ReverTra Ace^®^ qPCR RT Master Mix (Toyobo, Japan), and aliquots of 1-μl cDNA were amplified in a 20-μl PCR mixture that contained Platinum SYBR Green Realtime PCR Master Mix –Plus- (Toyobo, Japan) and gene-specific primers (Parkson Beijing) (Table 1) according to the manufacturer’s instructions. The reaction comprised an initial denaturation for 95°C for 2 min, 40 cycles with denaturing at 95°C for 15 s, and annealing and extension at 55°C for 15 s in each cycle, performed using a Step One Real-Time PCR System (Applied Biosystems, USA).

Primer sequences were designed on the basis of published gene sequences (NCBI and PubMed). GAPDH was chosen as an endogenous control for the study. The relative gene expression profiles of various samples were normalized to the corresponding GAPDH and analyzed using the 2^-△△CT^ approach. Each sample was assessed in triplicate.

**1.2. The biomimetic cell-free PCL-MECM scaffolds implantation in sheep model**

**1.2.1. Surgical procedure in the sheep model**

This study was conducted under the committee guidelines for animal experiments at Chinese PLA general hospital. In total, 20 sheep weighting 35 kg were randomly divided into the following four groups (*n* = 5 each group, Table 2) before operation: PCL-MECM scaffold, autograft, sham and control. The medial collateral ligament was cut to expose the posterior horn of the medial meniscus under anesthesia with intramuscular injections of Sumianxin II (0.1mL/kg) and ketamine (4mg/kg). A partial meniscectomy was then performed by resecting all but one third of the meniscus’ outer zone in right stifle joints of all sheep. Anatomically correct PCL-MECM scaffolds were implanted in the PCL-MECM scaffold group, and autologous menisci were implanted in the autograft group. Animals in the sham group only underwent exposure of the medial meniscus. Animals in the control group did not implant any tissues. The scaffolds and autologous menisci were sutured to the remaining meniscus. The capsule was then closed and the medial collateral ligament was reconstructed using resorbable sutures. After the operation, received intramuscular penicillin injections to prevent knee joint infections, and each sheep returned to its cage moving voluntarily. All sheep were euthanized and assessed at 3 months post-surgery.

**1.2.2. X-ray imaging and MRI**

The X-ray imaging and MRI scan was conducted the same as parts 2.3.2 previously.

**1.2.3. Macroscopic observations, histological and immunohistochemical analyses**

These parts were performed almost the same as parts 2.3.3 as previously, except that the corresponding distal femur and proximal tibia were decalcified in 10% EDTA solution for 56 days after being fixed in 4% PBS paraformaldehyde for 3 days.

**2. Results**

***2.1. Cell viability assessment***

The viability of meniscal fibrochondrocytes on various coated surfaces was assessed using the Live/Dead assay after 7 and 14 days culture (Figure S2). By day 7, there were more live cells (green spots) and fewer dead cells (red spots) on MECM and MECM/CS surface, in comparison with TCP and CS surface. After 14 days culture, more live cells adhered on the surfaces and fewer dead cells were observed than before. The live cells and dead cells distribution did not show any difference between four various coated surfaces after 14 days culture.

***2.2. Cell proliferation in MECM***

The cell proliferation characteristics of MECM were assessed using the CCK-8 quantitative assay after 1, 3, 5, 7 days of culture (Figure S3). The cell number increased from 1 to 7 days in both the MECM and control groups, whereas the cell proliferation capacity in the MECM group was significantly higher than that in the control group at 3, 5, and 7 days (P < 0.05).

***2.3. Cell* *adhesion and cellularity***

The DNA content of meniscal fibrochondrocytes cultured on various coated surfaces was quantified for 6 h, 7 and 14 days post-seeding. Then the mean DNA content of the samples was turned into cell numbers (Figure S 6a). After 6 hours cell seeding, the cells on the MECM surface showed the highest adherence (1.0 ± 0.1 x 10^4^ cells) than the cells on other surfaces, the cells on MECM/CS surface (8.4 ± 0.3 x 10^3^ cells) displayed slightly lower attachment than cells on MECM surface. The attachment of cells on TCP surface (6.7 ± 0.2 x 10^3^ cells) was similar to the CS surface (5.3±0.6 x 10^3^ cells). By day 7 and 14, the cell number of MECM surface remains the highest among the four various coated surfaces, likewise, the cell number of MECM/CS surface was slightly lower than cells on MECM surface, however, there was no significant difference between them (P > 0.05). The cell proliferation on TCP surface was comparable to CS surface (P > 0.05). Moreover, the cell proliferation trend was consistent with the cell morphology image (Figure 2 g).

***2.4. Total sGAG production***

The sGAG production of the fibrochondrocytes was assessed using sGAG content normalized to corresponding DNA content at 7 and 14 days post-seeding between various coated surfaces (Figure S 6b). At day 7, sGAG/DNA content on MECM/CS surface amounted to 2.1-fold content on CS surface (P < 0.05). The sGAG/DNA content on MECM surface was slightly lower than MECM/CS coated surface (P > 0.05). On the other hand, the sGAG/DNA content on TCP surface was higher than CS surface. At day 14, the sGAG/DNA content on various four surfaces all increased, whereas sGAG/DNA content on MECM surface was the highest content than the other surfaces, comparable to 1.7-fold content on CS surface (P < 0.05). On the other side, sGAG/DNA content on MECM/CS surface was lower than MECM coated surface, however, there was no significant difference between them (P > 0.05).

***2.5. Total collagen production***

Similarly, the collagen production of cells was estimated by total collagen normalized to corresponding DNA content at the same time points between various coated surfaces (Figure S 6c). At day 7, MECM surface had the highest Col/DNA content, which was 5.8-fold and 6.4-fold greater than those from TCP surface and CS surface (P < 0.05), respectively. The Col/DNA on MECM/CS surface was slightly lower than MECM surface (P > 0.05). The Col/DNA content after 14 days culture increased on four various surfaces. The Col/DNA content on MECM and MECM/CS surface remained higher than the other two surfaces (P < 0.05), moreover, there is no significant difference of Col/DNA content between them (P > 0.05).

***2.6. Gene expression analysis***

To explore the effect of the various coated surfaces on the mRNA levels of dedifferentiated meniscal fibrochondrocytes, RT-qPCR analysis was used to evaluate the expression of selected genes, in comparison with the P3 fibrochondrocytes before seeding. Only collagen II was up-regulated in fibrochondrocytes on the MECM and MECM/CS surfaces (Figure S8) at day 7 or 14. At day 7, the collagen II mRNA level increased by 6.5-fold (P <0.05) on the MECM surface and 4.5-fold (P <0.05) on the MECM/CS surface. At day 14, collagen II mRNA expression on the MECM surface continued was increased by 11.5-fold (P <0.05), compared to 6.8-fold on the MECM/CS surface (P <0.05).

***2.7. The PCL-MECM scaffolds promoted meniscus in situ regeneration in sheep model post 3 months implantation***

Similarly, we implanted the PCL-MECM scaffold in sheep model that had undergone partial meniscectomy (Figure S20). In the PCL-MECM scaffold group, some neo-menisci were observed and well covered the corresponding knee cartilage at 3 months post-implantation (Figure S21a, 21c). There is remaining the one third outside meniscus in control group. In terms of cartilage protection, there was little obvious damage to the cartilage surface in the PCL-MECM scaffold group and autograft group, whereas obvious cartilage defect in the control group. Histological staining confirmed that the neo-menisci in the PCL-MECM scaffold group also demonstrated meniscus-like heterogeneous staining characteristics (Figure S21b, S22). On the one hand, there were numerous round-shaped chondrocyte-like cells in the inner region of the neo-menisci, while there were abundant elongated fibroblast-like in the outer region of the neo-menisci at 3 months post-implantation. On the other hand, the collagen fiber of the neo-menisci displayed an oriented formation at 3 months post-implantation. Moreover, the histological score in the PCL-MECM scaffold group was significantly better than that in the meniscectomy group, and it was similar to that both in the meniscus autograft and sham groups (Figure S21d). Similarly, there were also some residual PCL in the inner region and the outer region of the neo-menisci at 3 months post-implantation, while no obvious MECM in the neo-menisci. The chondroprotective effect of neo-meniscus was evaluated by histological examination and Mankin scores. Histological examination revealed that the cartilage surface was better preserved in the PCL-MECM scaffold group than that in the control group at 3 months post-implantation. Meanwhile, Mankin scores in the PCL-MECM scaffold group were significantly better than those of the control group at 3 months post-implantation (Figure S21e, S21f, S21g, S21h).

***2.8. The X-ray and MRI assessment after PCL-MECM scaffolds implantation in sheep model post 3 months implantation***

The knee X-ray examination revealed obvious knee joint degeneration in the control group. Similarly, the Kellgren-Lawrence scores in control group were higher than those in the other three groups. There was no obvious cartilage degeneration in the PCL-MECM scaffold group (Figure S21i, S21j). On the one hand, MRI examination displayed obvious synovial inflammation signals and thinner cartilage thickness in the control group, whereas no obvious inflammatory signals in the PCL-MECM scaffold group. On the other hand, there were distinct neo-meniscus signals in the PCL-MECM scaffold group, whereas only inflammatory signals were observed in the control groups. Similarly, the knee MRI results showed lower WORMS scores in the PCL-MECM scaffold group than that in the control group at 3 months post-implantation (Figure S23).


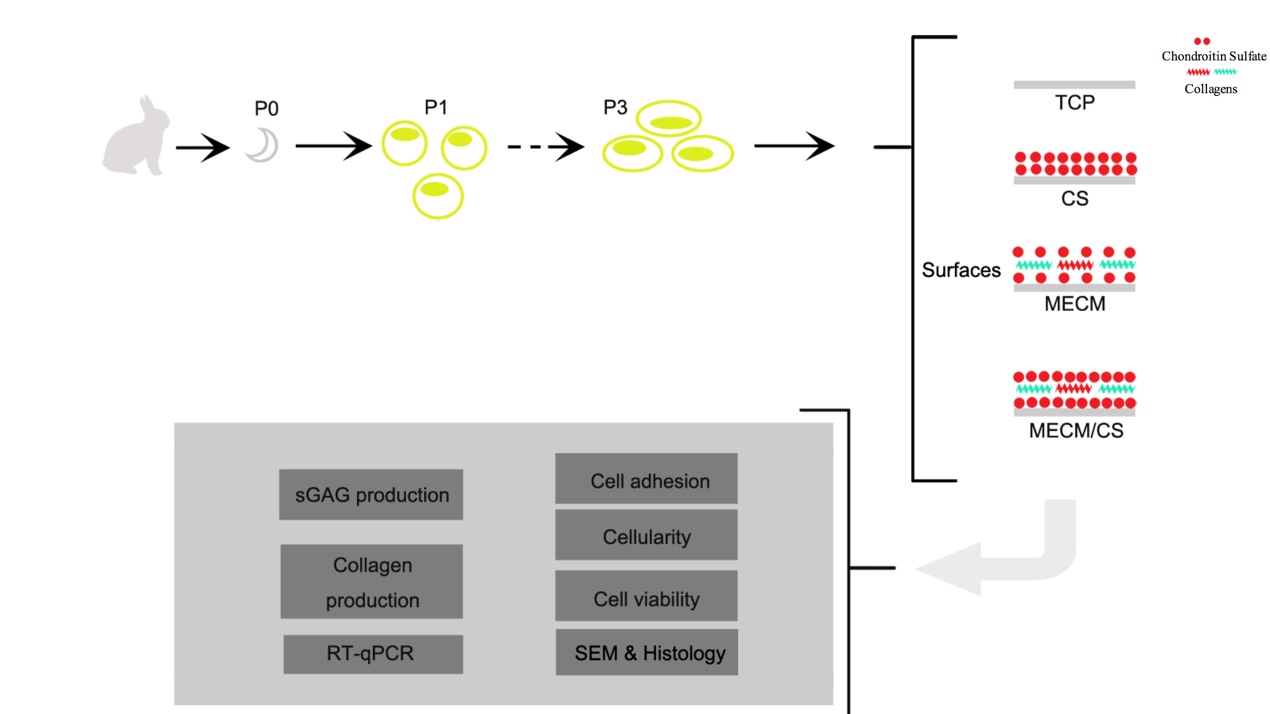


**Figure S1.** A schematic diagram illustrated the experimental outline for meniscal fibrochondrocytes on the various coated surfaces.


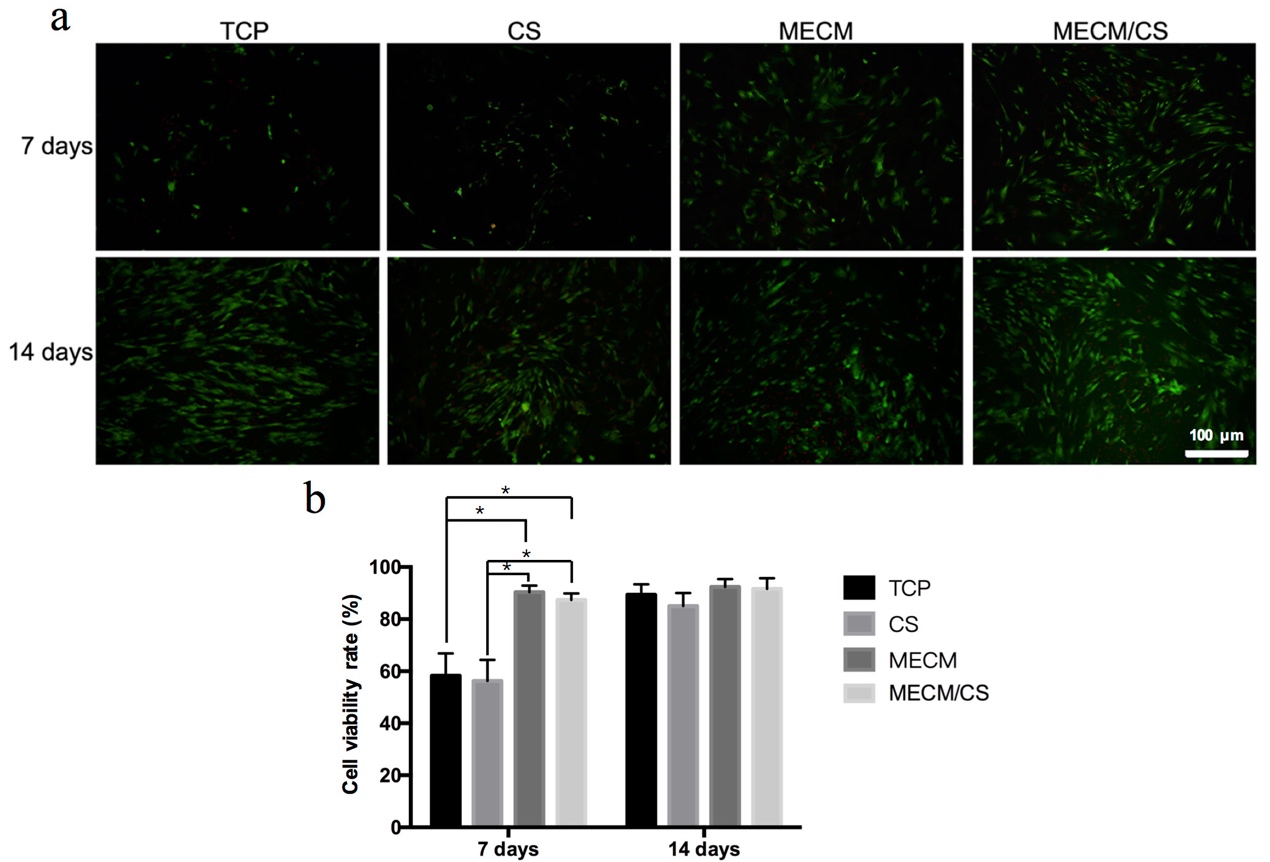


**Figure S2.** Cellular viability of meniscal fibrochondrocytes on various coated surfaces after 7 and 14 days culture. All experiments were independently repeated in triplicate. Error bars represent standard deviation (* P < 0.05).


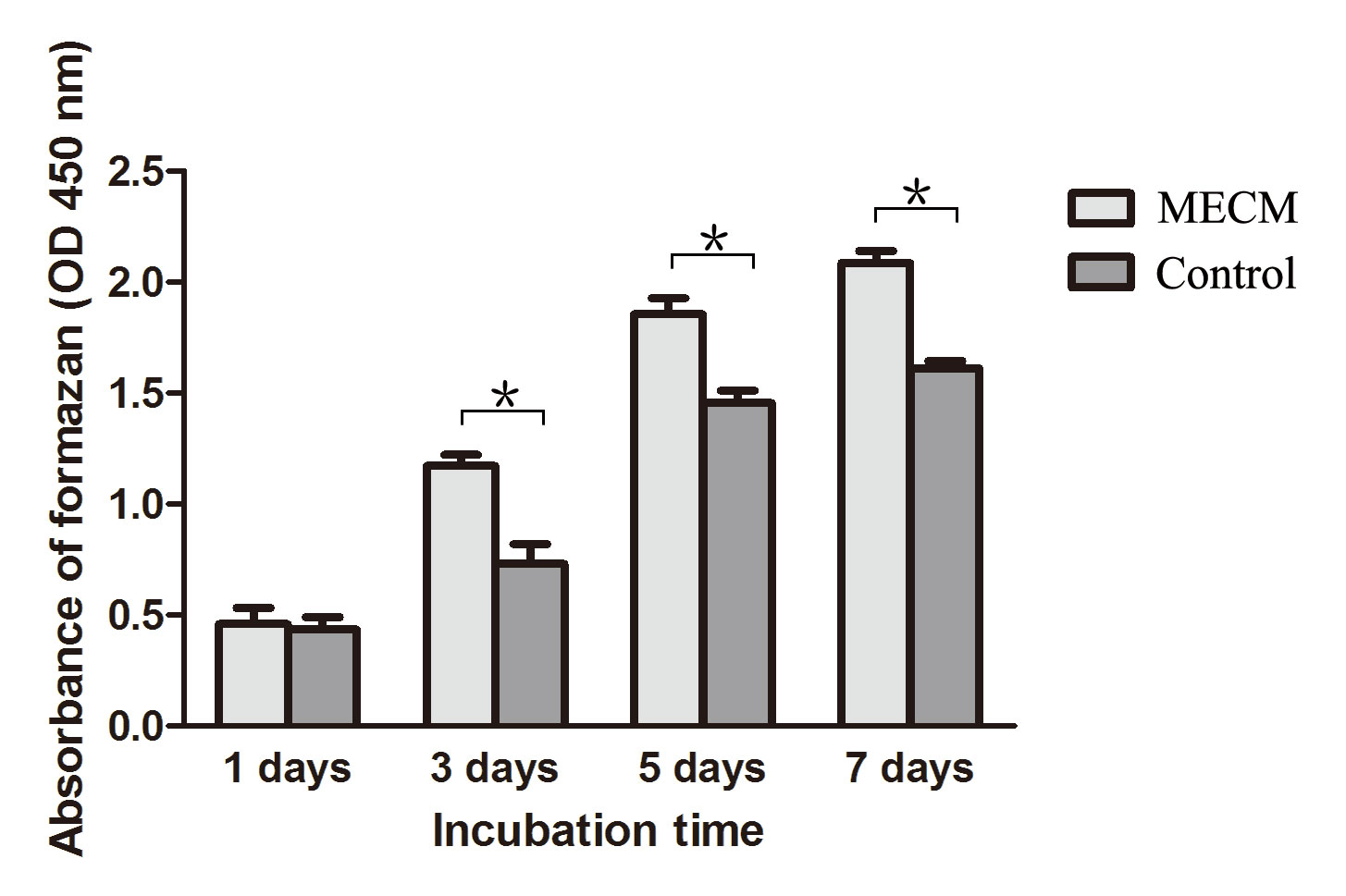


**Figure S3.** The cell proliferation characteristics of MECM was assessed using CCK-8 assay. All experiments were independently repeated in sextuplicate. Error bars represent standard deviation (* P < 0.05).


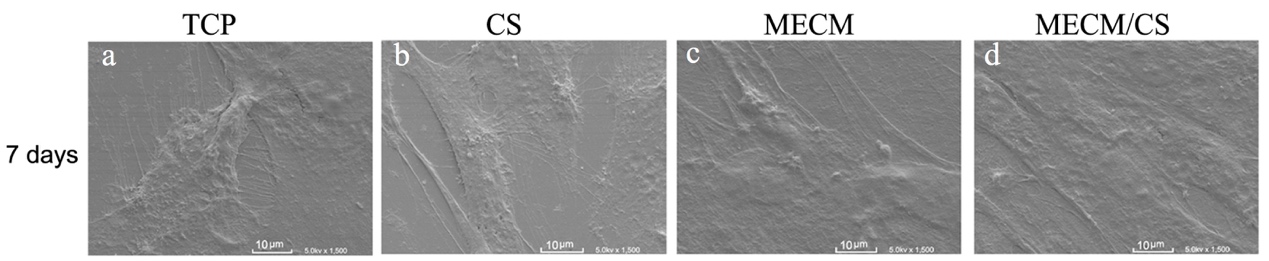


**Figure S4.** SEM assessment of meniscal fibrochondrocytes on various coated surfaces after 7 days culture.


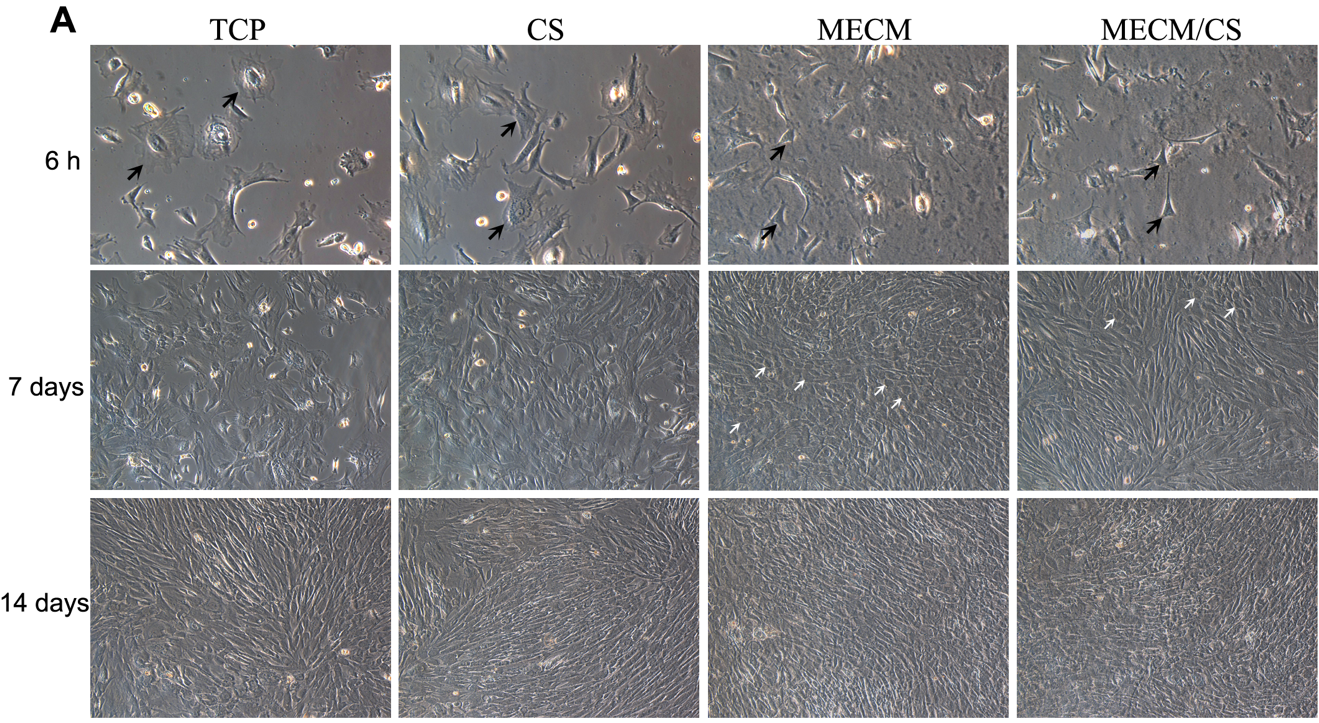


**Figure S5.** Cellular appearance of meniscal fibrochondrocytes on various coated surfaces.

**
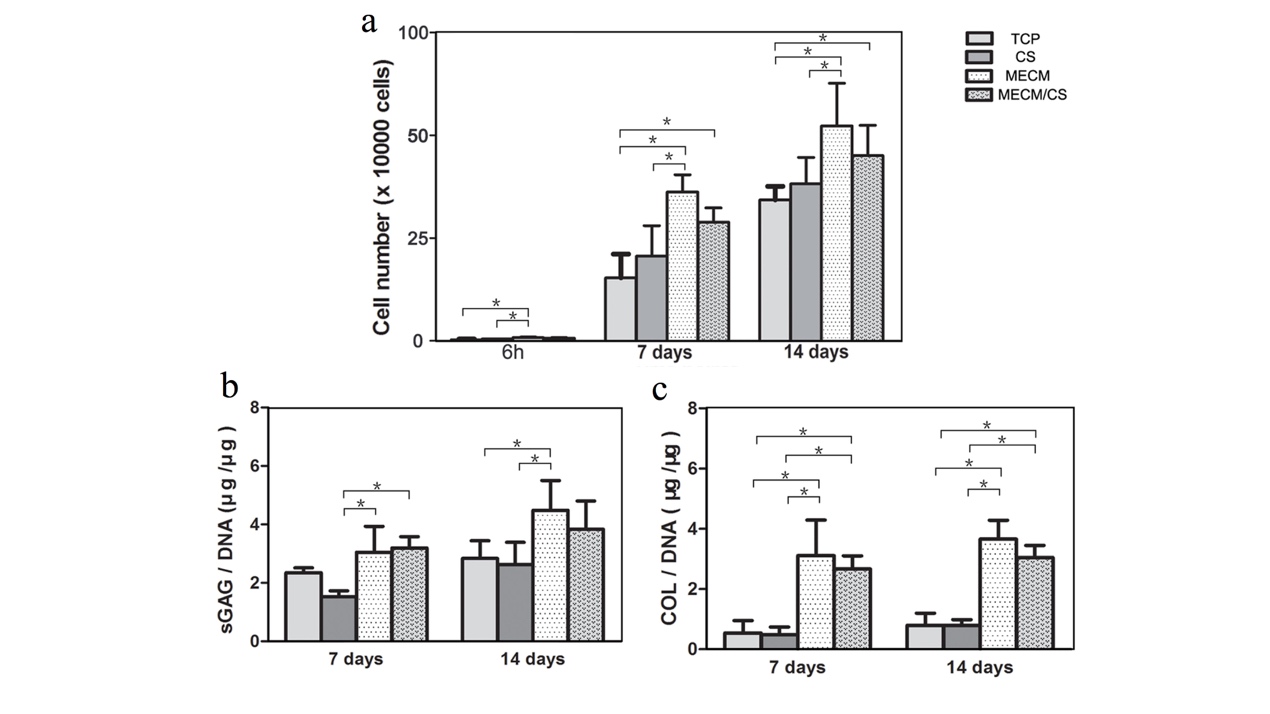
**

**Figure S6.** Cellularity, matrix production of meniscal fibrochondrocytes on various coated surfaces after 7 and 14 days culture. All experiments were independently repeated in triplicate. Error bars represent standard deviation (* P < 0.05).


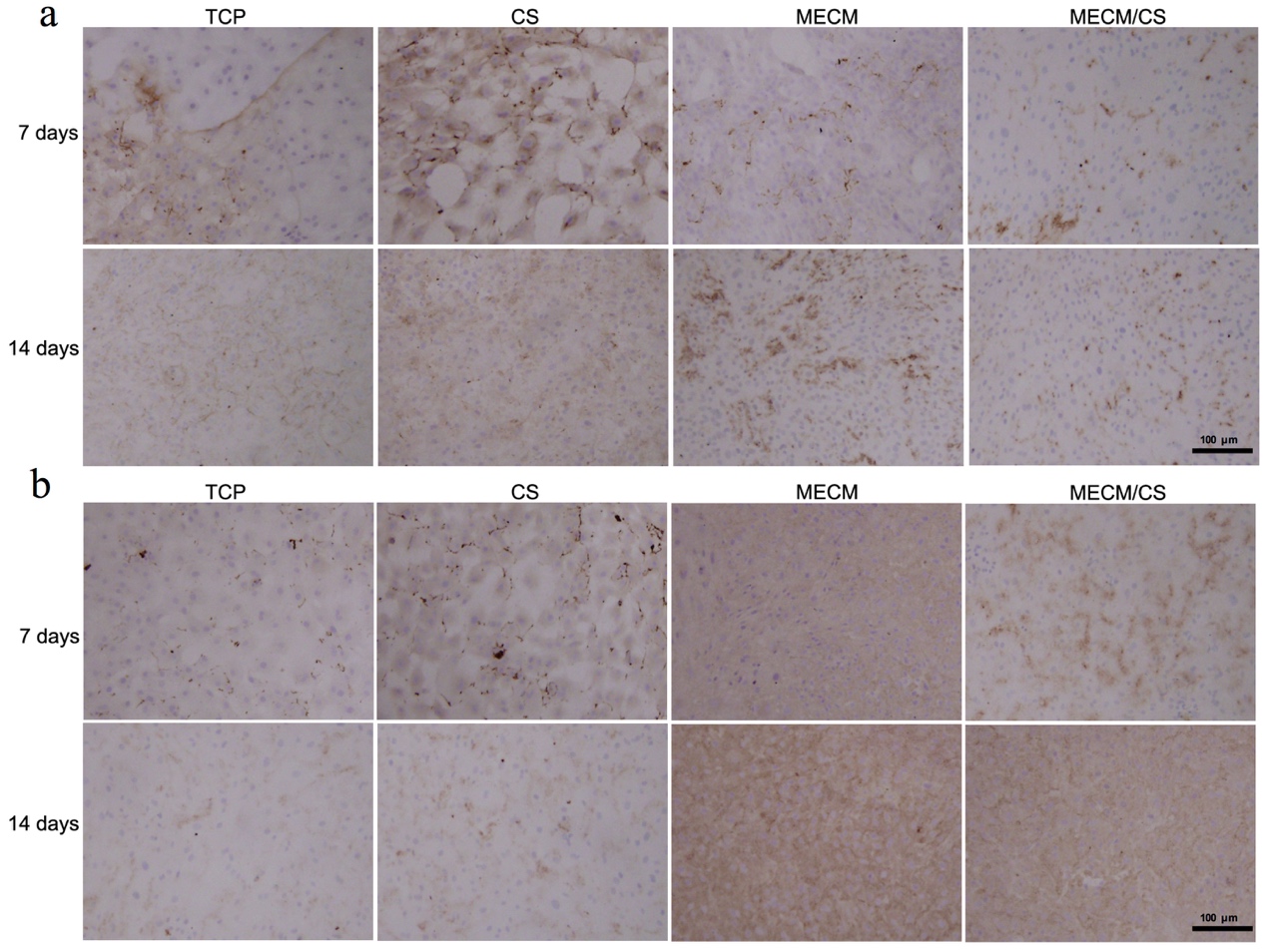


**Figure S7.** Immumohistochemical staining of meniscal fibrochondrocytes on various coated surfaces after 7 and 14 days culture.


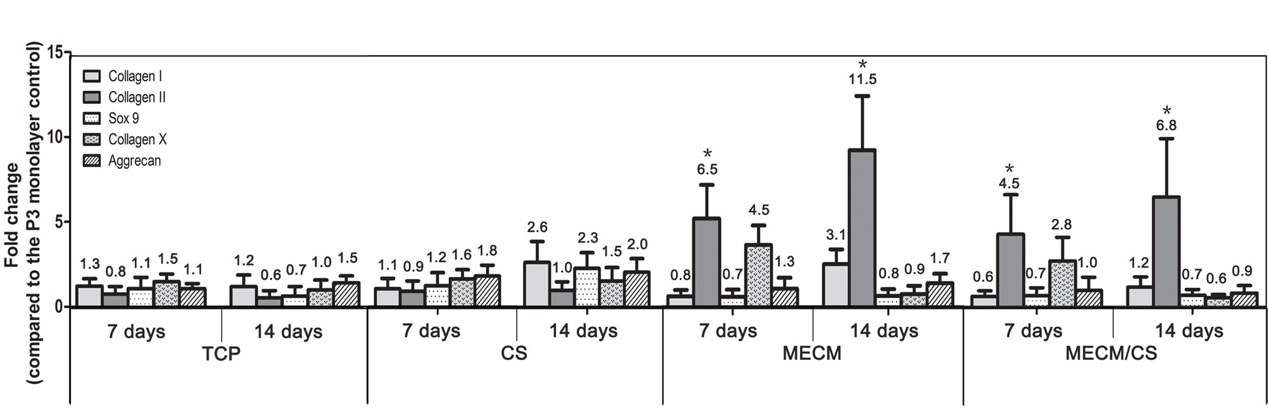


**Figure S8.** Comparative gene expression analysis of meniscal cells on various coated surfaces after 7 and 14 days culture. All experiments were independently repeated in triplicate. Error bars represent standard deviation (* P < 0.05).

**
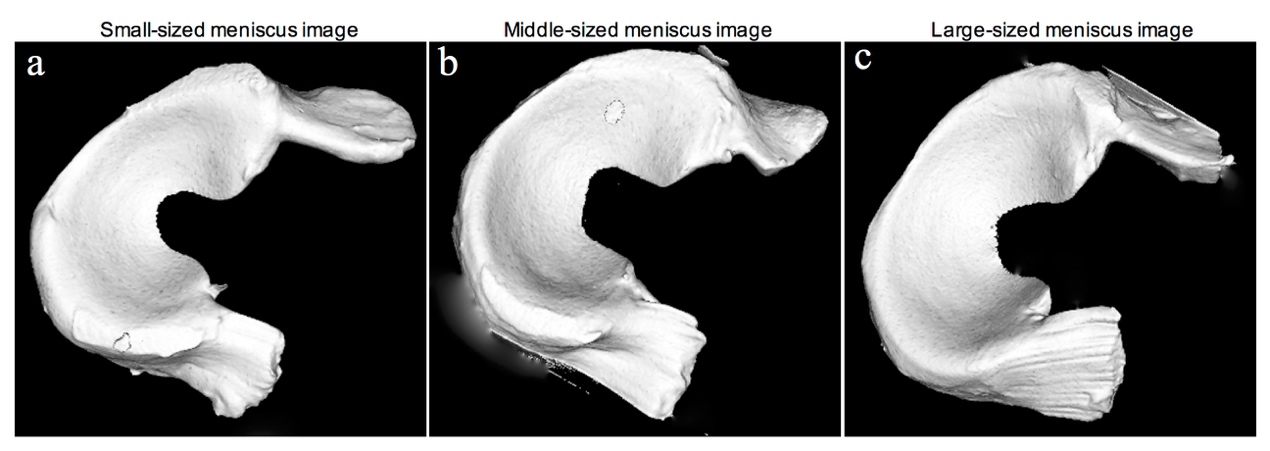
**

**Figure S9.** Micro-CT image of various type sheep meniscus.

**
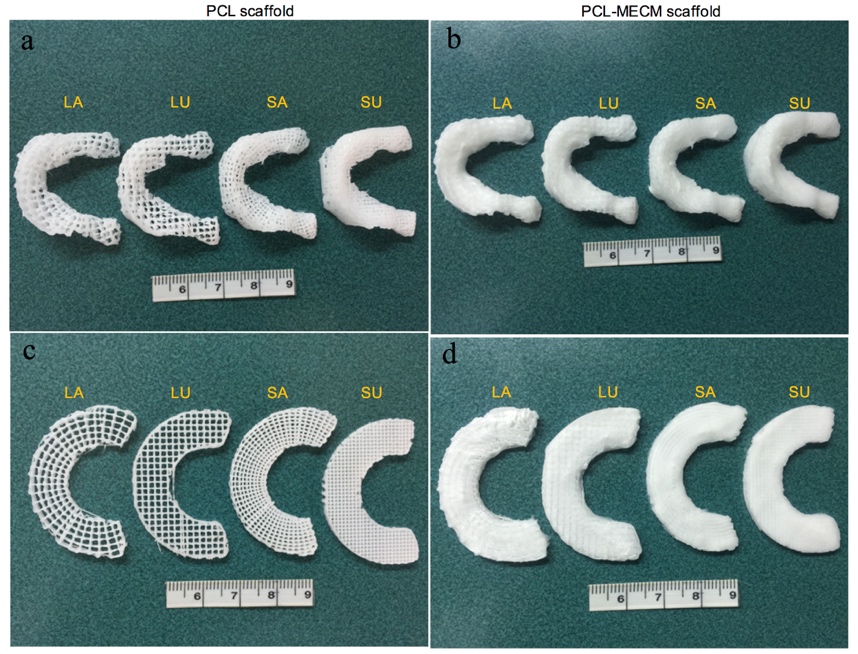
**

**Figure S10.** Macroscopic image of PCL scaffold and PCL-MECM scaffold.

**
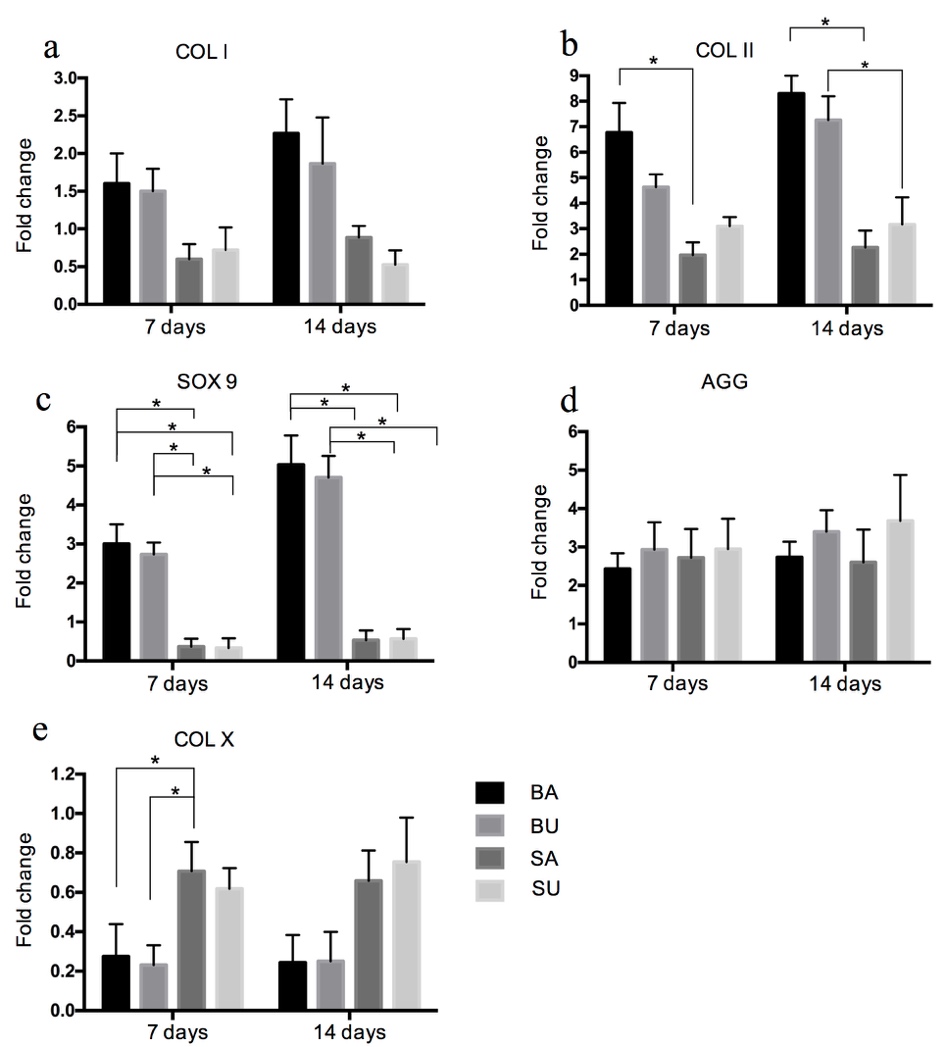
**

**Figure S11.** Comparative gene expression analysis of meniscal cells on various PCL-MECM scaffolds after 7 and 14 days culture. All experiments were independently repeated in triplicate. Error bars represent standard deviation (* P < 0.05).

**
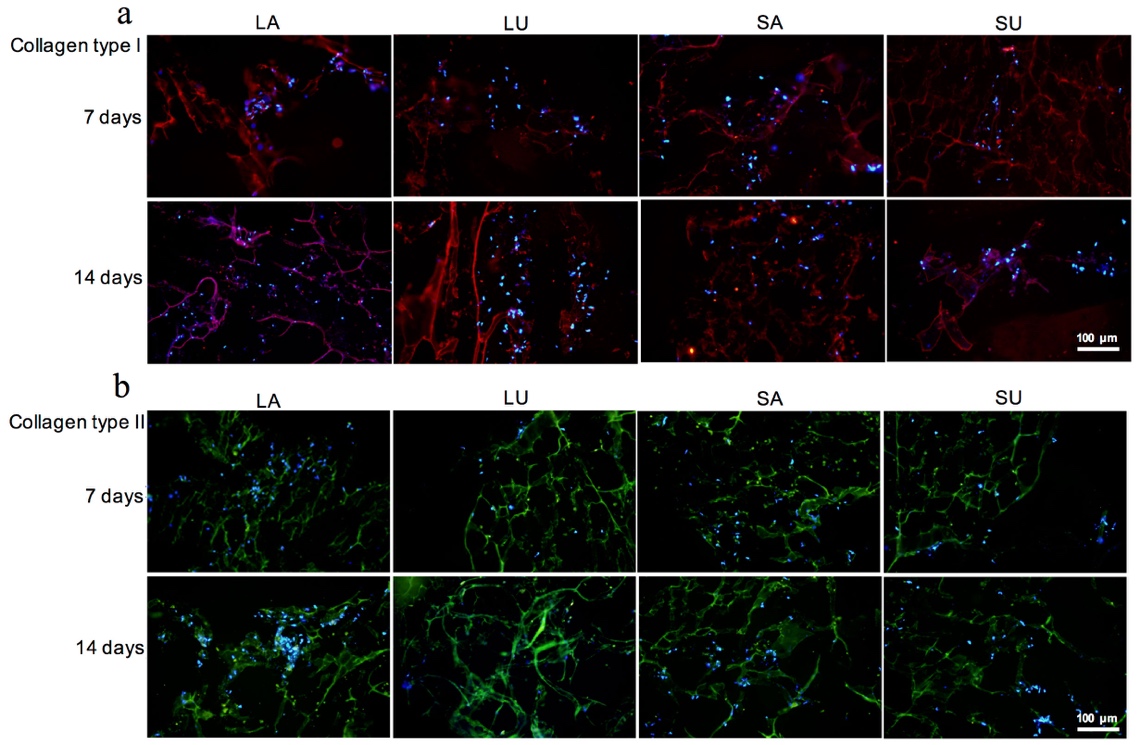
**

**Figure S12.** Immumofluorescence staining of meniscal fibrochondrocytes on various PCL-MECM scaffolds after 7 and 14 days culture.

**
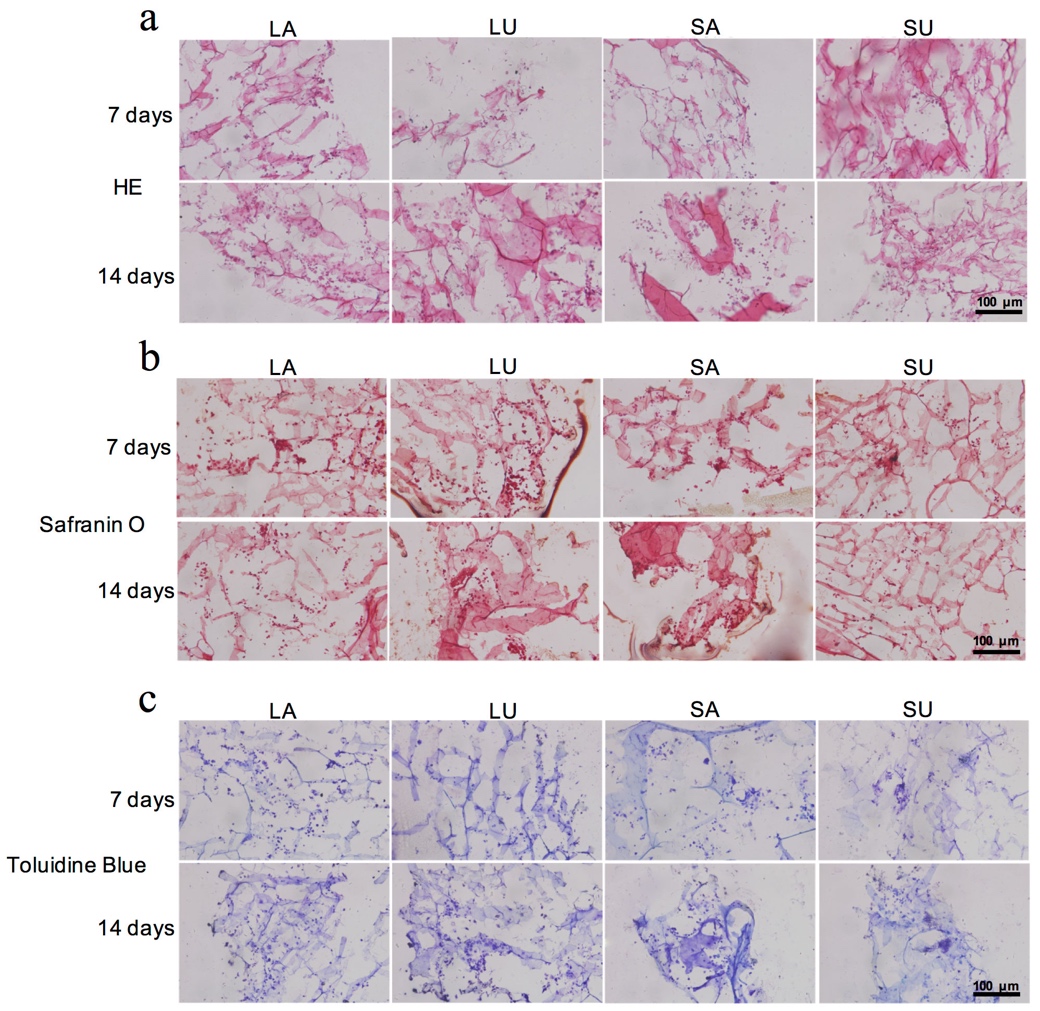
**

**Figure S13.** HE, Safranin O, and toluidine blue staining of the meniscal fibrochondrocytes and PCL-MECM scaffolds constructs after 7 and 14 days culture.

**
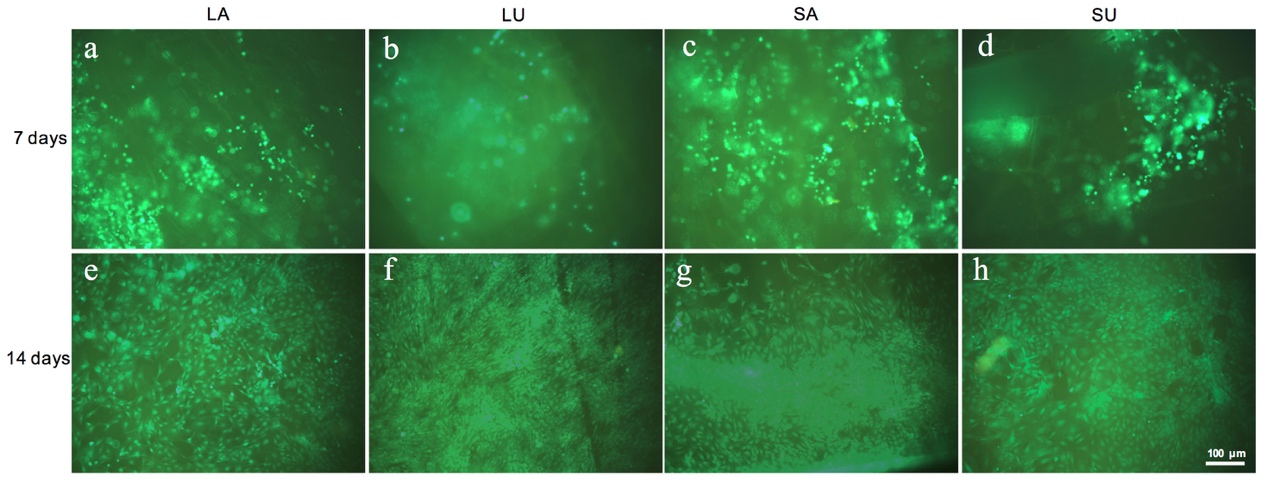
**

**Figure S14.** Confocal microscopy image of the GFP rat meniscal fibrochondrocytes after 7 and 14 days on various PCL-MECM scaffolds.

**
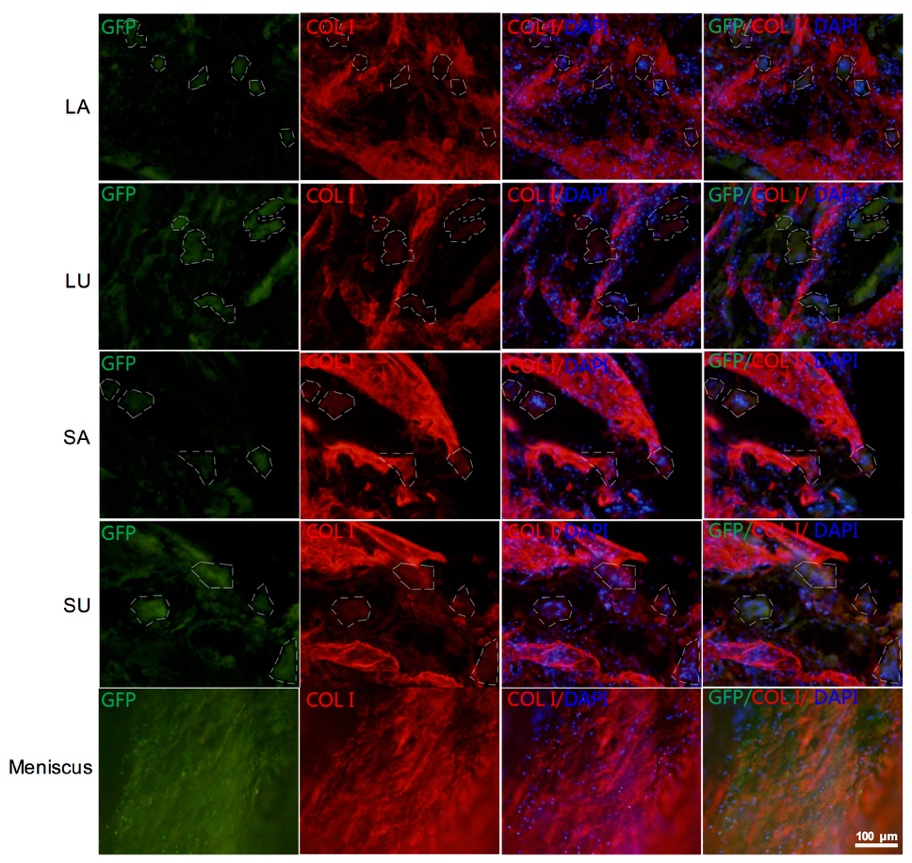
**

**Figure S15.** Immumofluorescence staining of collagen type I in the rat GFP meniscal fibrochondrocytes and PCL-MECM scaffolds constructs in nude rats 4 weeks after subcutaneous implantation**.**

**
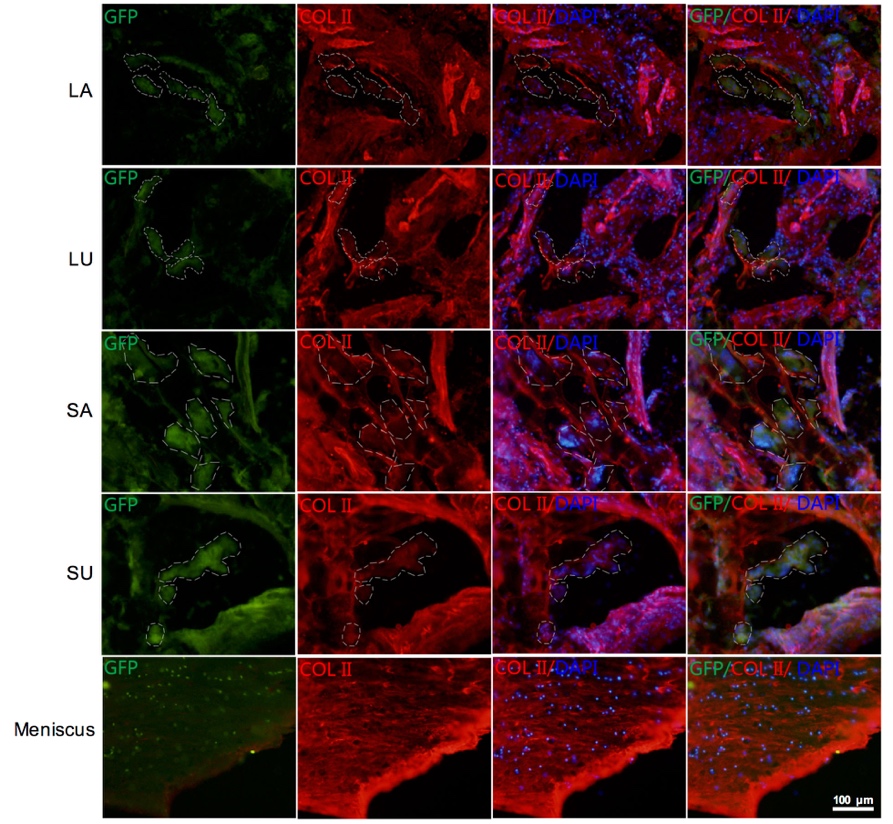
**

**Figure S16.** Immumofluorescence staining of collagen type II in the rat GFP meniscal fibrochondrocytes and PCL-MECM scaffolds constructs in nude rats 4 weeks after subcutaneous implantation**.**

**
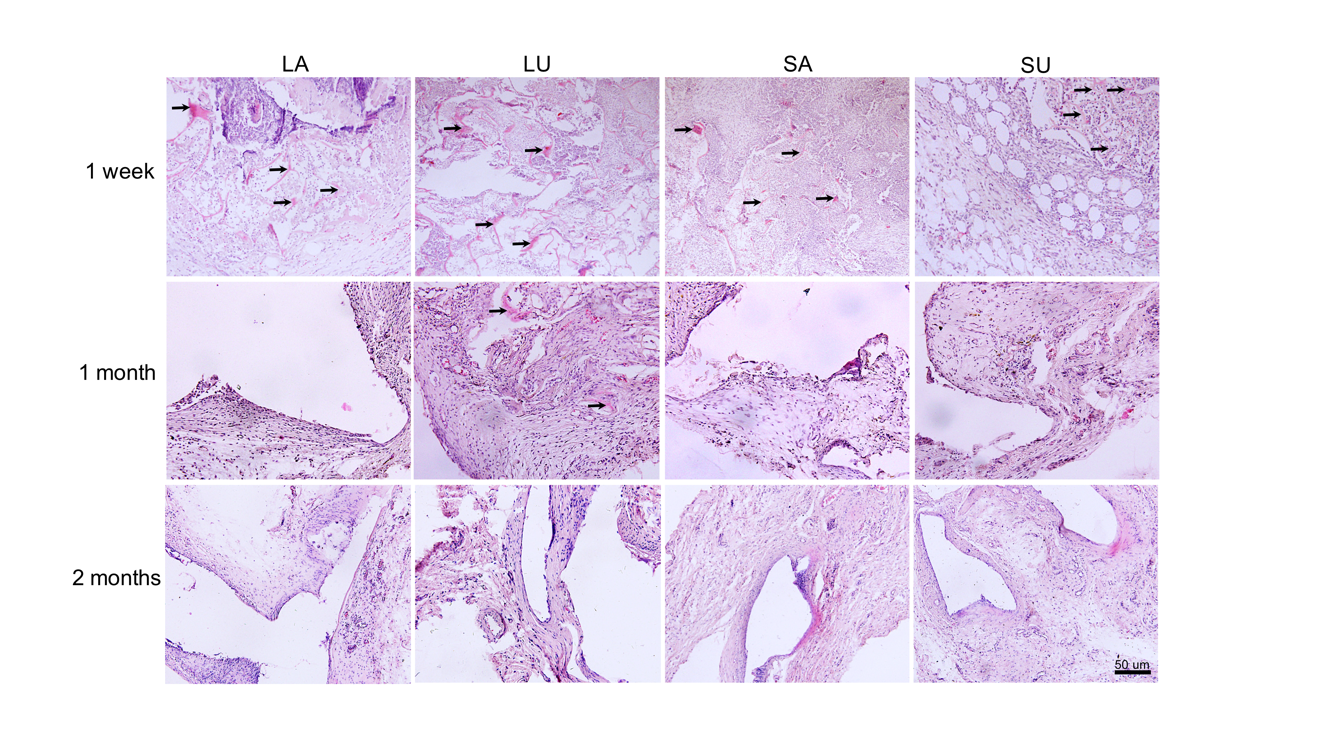
**

**Figure S17.** HE staining of the PCL-MECM scaffolds after 1 week, 1 month and 2 months rat subcutaneous tissues implantation. (PCL: White void spaces, MECM: Black arrows indicate)

**
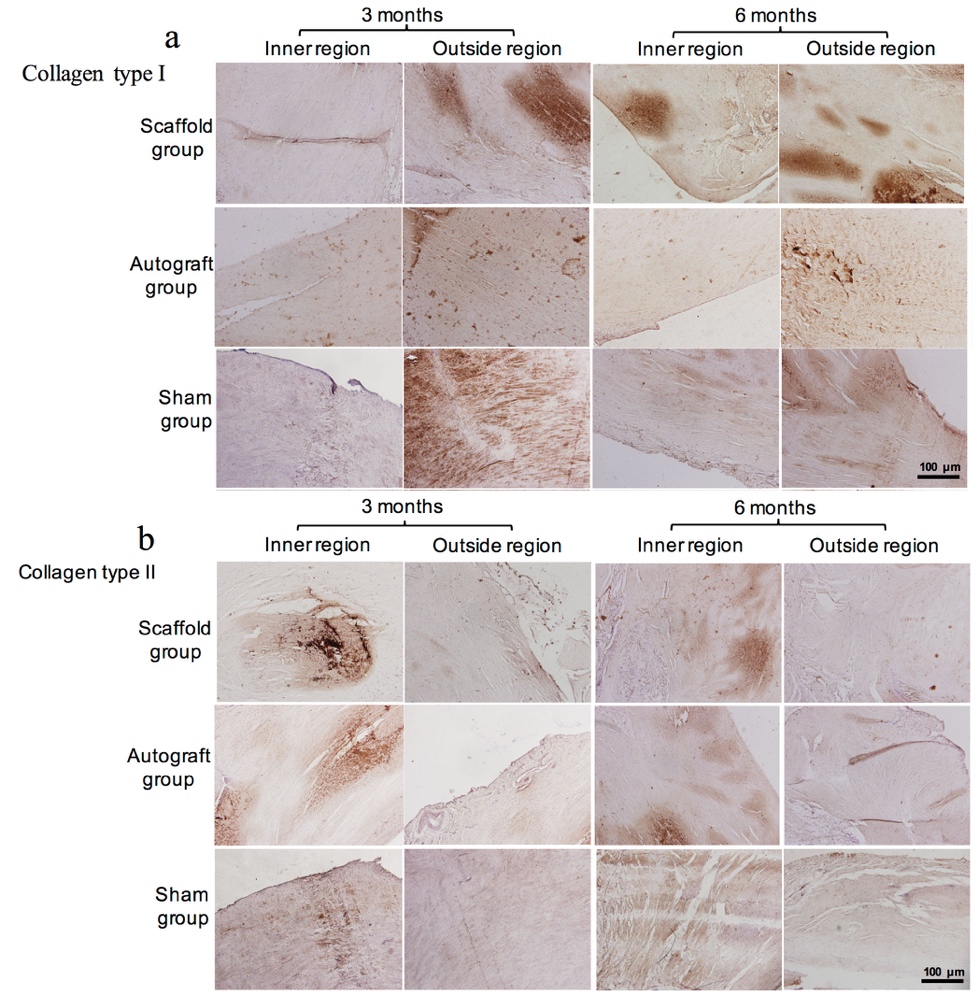
**

**Figure S18.** Immumohistochemical staining of neo-menisci in a rabbit meniscus injury repair model 3 and 6 months post-surgery.

**
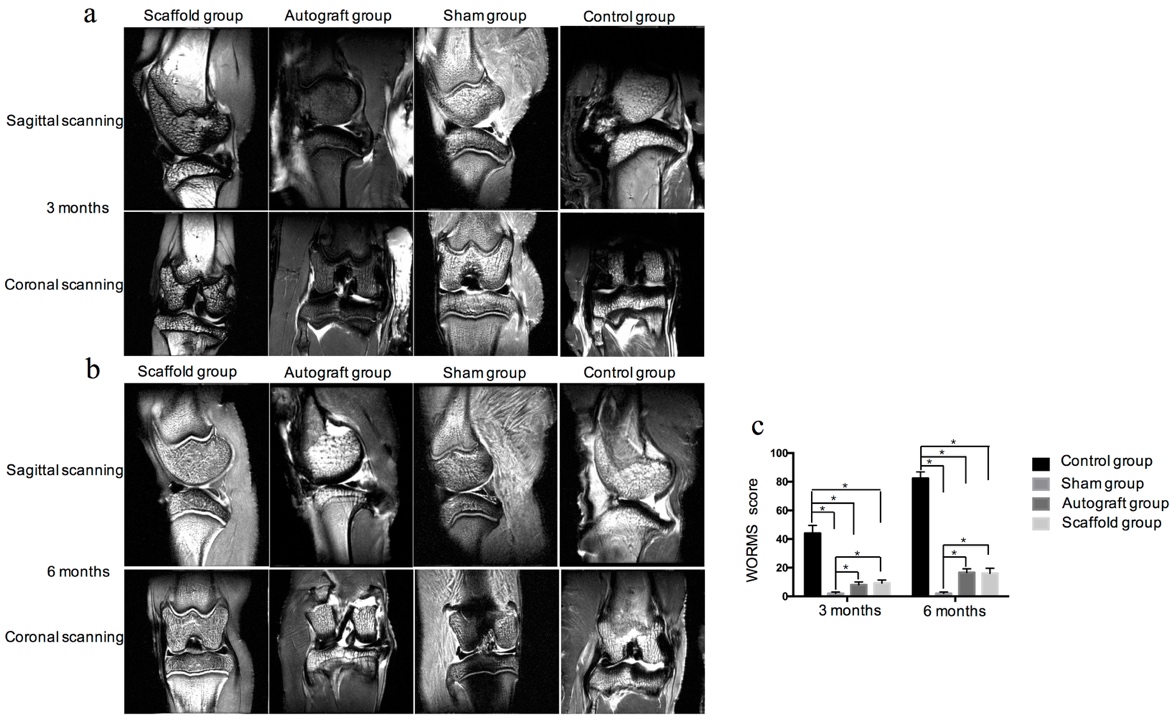
**

**Figure S19.** MRI images and WORMS scores in the various groups 3 and 6 months post-surgery in a rabbit meniscus injury repair model. All experiments were independently repeated in triplicate. Error bars represent standard deviation (* P < 0.05)

**
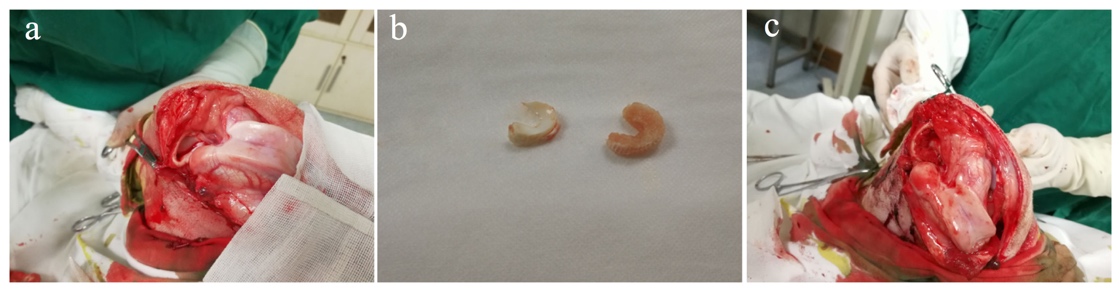
**

**Figure S20.** PCL-MECM scaffolds implantation process in sheep model.


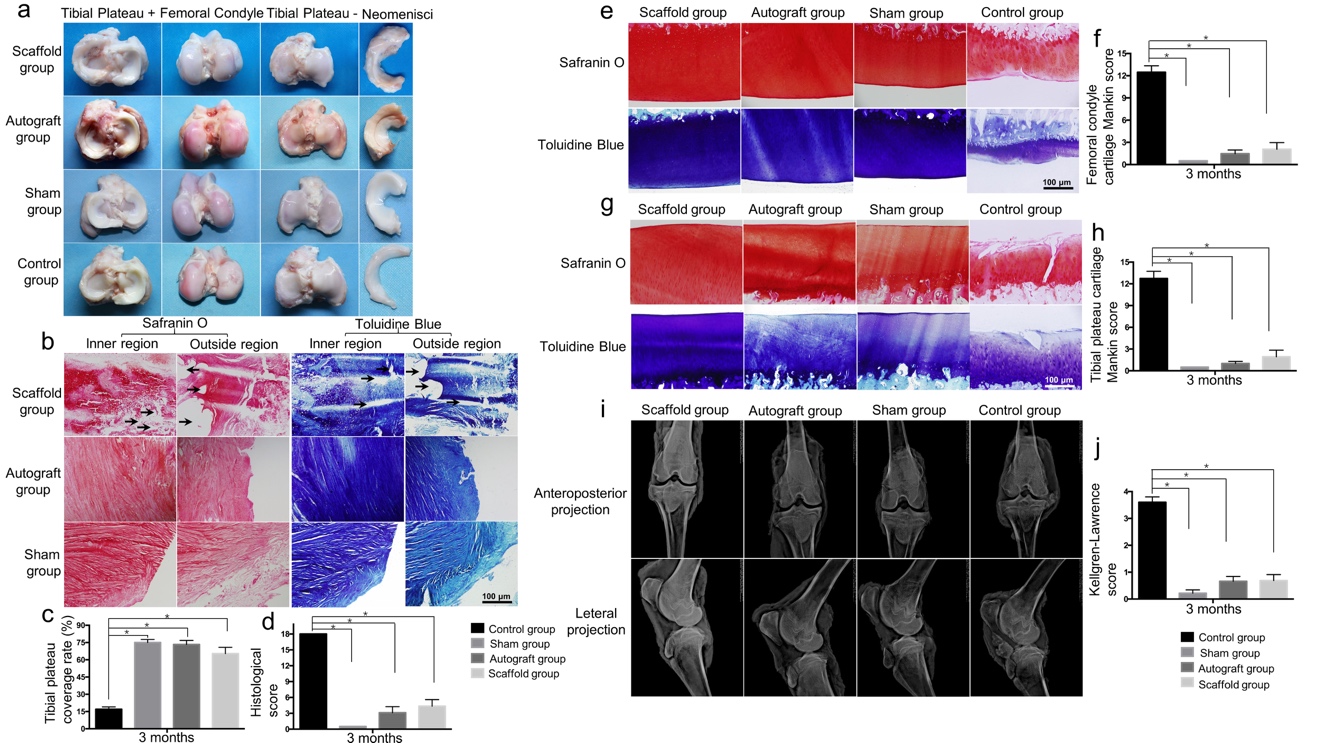


**Figure S21.** Comprehensive assessment of the neo-menisci in a sheep meniscus injury repair model 3 months post-surgery. a) Macroscopic analysis of neo-menisci and articular cartilage of the corresponding tibial plateau and femoral condyles. b) Histological staining of neo-menisci (Black arrows indicate degraded PCL). c) Tibial plateau coverage rate of neo-menisci. d) Histological scores of the neo-menisci. e), f), g) h) Histological staining and scores of articular cartilage in the corresponding tibial plateau and femoral condyles, respectively. i), j) X-ray assessment and scores of the corresponding knee joints. All experiments were independently repeated in triplicate. Error bars represent standard deviation (* P < 0.05).

**
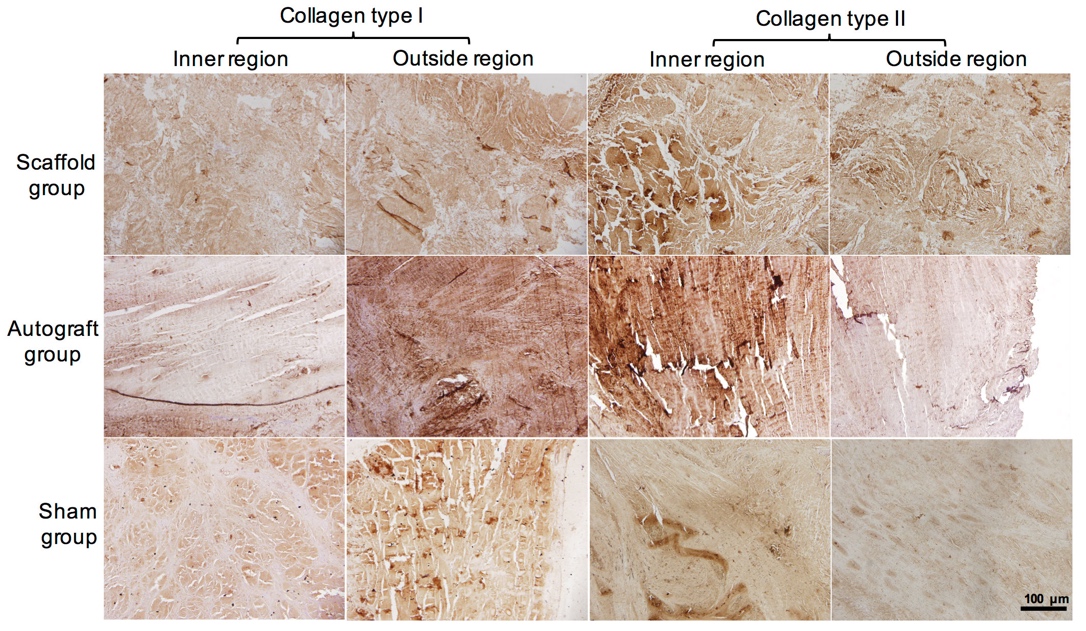
**

**Figure S22.** Immumohistochemical staining of neo-menisci in a sheep meniscus injury repair model 3 months post-surgery.

**
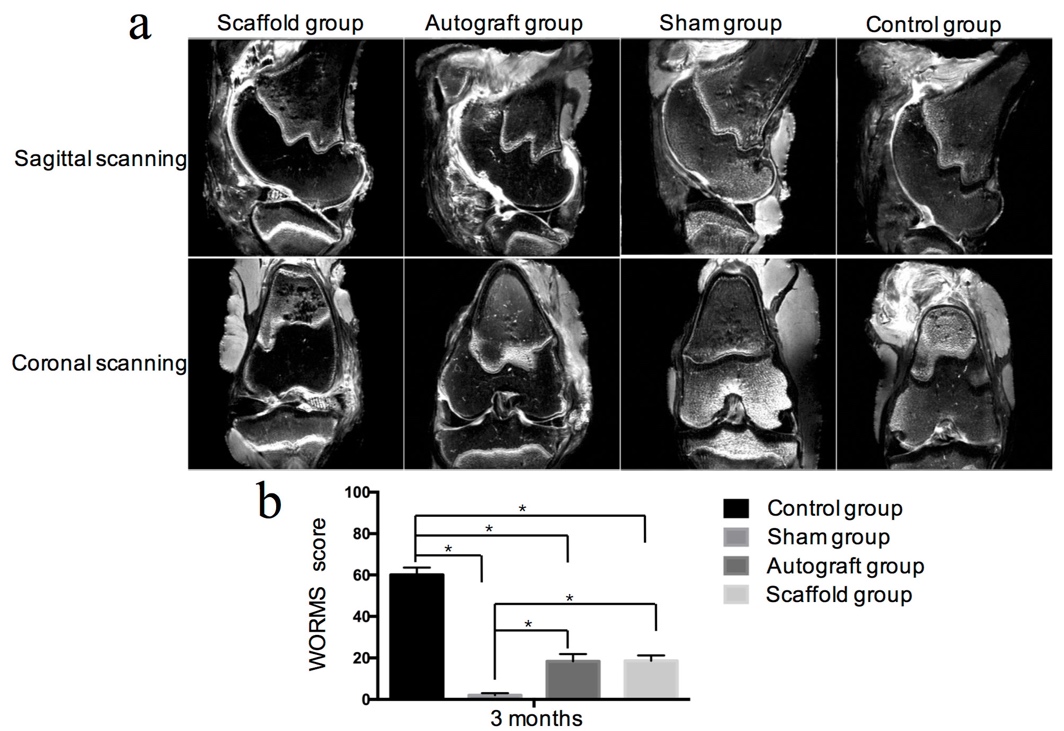
**

**Figure S23.** MRI images and WORMS scores in the various groups 3 months post-surgery in a sheep meniscus injury repair model. All experiments were independently repeated in triplicate. Error bars represent standard deviation (* P < 0.05)

**Reference**

[1] Y.J. Kim, R.L. Sah, J.Y. Doong, A.J. Grodzinsky, Fluorometric assay of DNA in cartilage explants using Hoechst 33258, Analytical biochemistry 174(1) (1988) 168-76.
